# Temporally distinct transcriptional regulation of myocyte dedifferentiation and Myofiber growth during muscle regeneration

**DOI:** 10.1186/s12864-017-4236-y

**Published:** 2017-11-09

**Authors:** Ke’ale W. Louie, Alfonso Saera-Vila, Phillip E. Kish, Justin A. Colacino, Alon Kahana

**Affiliations:** 1 Department of Ophthalmology and Visual Sciences, Kellogg Eye Center, University of Michigan, 1000 Wall St, Ann Arbor, MI 48105 USA; 2 Department of Biologic and Materials Sciences, School of Dentistry, University of Michigan, 1011 N. University, Ann Arbor, MI 48109 USA; 3 Department of Environmental Health Sciences, School of Public Health, University of Michigan, 1415 Washington Heights, Ann Arbor, MI 48109 USA; 4 University of Michigan Comprehensive Cancer Center, 1500 E Medical Center Dr, Ann Arbor, MI 48109 USA; 5 Department of Nutritional Sciences, School of Public Health, University of Michigan, 1415 Washington Heights, Ann Arbor, MI 48109 USA

**Keywords:** Transcriptome, RNA-sequencing, Cell reprogramming, Zebrafish, Stem cell, Polycomb, Fibronectin, Chromatin, Epigenetic

## Abstract

**Background:**

Tissue regeneration requires a series of steps, beginning with generation of the necessary cell mass, followed by cell migration into damaged area, and ending with differentiation and integration with surrounding tissues. Temporal regulation of these steps lies at the heart of the regenerative process, yet its basis is not well understood. The ability of zebrafish to dedifferentiate mature “post-mitotic” myocytes into proliferating myoblasts that in turn regenerate lost muscle tissue provides an opportunity to probe the molecular mechanisms of regeneration.

**Results:**

Following subtotal excision of adult zebrafish lateral rectus muscle, dedifferentiating residual myocytes were collected at two time points prior to cell cycle reentry and compared to uninjured muscles using RNA-seq. Functional annotation (GAGE or K-means clustering followed by GO enrichment) revealed a coordinated response encompassing epigenetic regulation of transcription, RNA processing, and DNA replication and repair, along with protein degradation and translation that would rewire the cellular proteome and metabolome. Selected candidate genes were phenotypically validated in vivo by morpholino knockdown. Rapidly induced gene products, such as the Polycomb group factors Ezh2 and Suz12a, were necessary for both efficient dedifferentiation (i.e. cell reprogramming leading to cell cycle reentry) and complete anatomic regeneration. In contrast, the late activated gene fibronectin was important for efficient anatomic muscle regeneration but not for the early step of myocyte cell cycle reentry.

**Conclusions:**

Reprogramming of a “post-mitotic” myocyte into a dedifferentiated myoblast requires a complex coordinated effort that reshapes the cellular proteome and rewires metabolic pathways mediated by heritable yet nuanced epigenetic alterations and molecular switches, including transcription factors and non-coding RNAs. Our studies show that temporal regulation of gene expression is programmatically linked to distinct steps in the regeneration process, with immediate early expression driving dedifferentiation and reprogramming, and later expression facilitating anatomical regeneration.

**Electronic supplementary material:**

The online version of this article (10.1186/s12864-017-4236-y) contains supplementary material, which is available to authorized users.

## Background

The goal of regenerative medicine is to replace lost tissue with fully functional regenerated tissue following trauma or disease. Given their highly-specialized structure and function, skeletal muscles are particularly prone to tissue loss following disease or injury, with devastating effects on function and quality of life [1, 2]. Although humans do not have extensive muscle regeneration capabilities that persist beyond the embryonic stage, tissue regeneration is observed in other vertebrate lineages and has been well studied in both amphibian and piscine models [3–5].

Regeneration of lost muscle requires the generation of an adequate number of myocytes to match the lost tissue and provide replacement function. Accumulation of such a regenerative cell mass can occur via proliferative expansion of resident tissue stem cells (i.e. satellite cells; [6, 7]), recruitment of cells from outside the damaged tissue area, or dedifferentiation of residual cells into progenitor cells capable of robust proliferation and redifferentiation [8, 9]. Blastema formation is observed in both urodele amphibian leg regeneration models and fish fin regeneration models [10, 11] and appears to utilize all three regenerative pathways [3]. On the other hand, regeneration of zebrafish retina, bone, cartilage, heart, liver, and extraocular muscles (EOMs) primarily utilizes dedifferentiation of residual cells [8, 9, 12–15]. The ability to reprogram “post-mitotic” cells into dedifferentiated proliferating progenitor cells represents a particularly potent approach for adult tissue regeneration and an alternative or complementary method to stem cell-based techniques [16].

Zebrafish EOMs regenerate rapidly following tissue loss, with myocyte dedifferentiation leading to myoblast proliferation by 20–24 h post injury (hpi), and anatomic muscle regeneration followed by functional recovery within 8–10 days post injury (dpi) [8]. In order to understand the biological events that lead to reprogramming of a highly-specialized cell such as a “post-mitotic” multinucleated syncytial myocyte, we focused on temporally altered transcriptional events that occur at time points (9 and 18 hpi) ending just prior to cell cycle reentry by dedifferentiated myoblasts, which is observed at 20–24 hpi [8]. Aided by the relatively homogenous population of dedifferentiating cells within the remaining muscle and utilizing these pre-proliferative time points, we performed a comprehensive transcriptome analysis. We were particularly interested in the broad, network-like interactions expected to occur within the dynamic biological landscape of reprogramming cells [8, 17, 18].

We report that following a partial myectomy, genes encoding muscle differentiation and morphogenic programs are downregulated over time. At the same time, cellular metabolism is rewired to accommodate the new needs, while new protein synthesis, along with lysosomal and ubiquitin ligated proteolysis, is upregulated to reshape the cellular proteome. Programs related to DNA replication, repair, and chromosome condensation are similarly upregulated and prepare the cell to reenter the cell cycle. The rapid activation of epigenetic regulators of transcription likely reflects the genomic regulatory changes driving myocyte dedifferentiation. Based on this functional analysis, we formulated and tested the hypothesis that early-activated genes would be important for the early events of myocyte cellular reprogramming and dedifferentiation, while late-activated genes would regulate the later anatomic regeneration of the muscle but would not affect the initial reprogramming events. The results of our in vivo experiments reveal that myocyte dedifferentiation appears to depend on transcriptional and epigenetic regulation, with key roles for early-activated genes such as the Polycomb group factors *ezh2* and *suz12a*. On the other hand, late-activated genes, such as *fn1* (encoding fibronectin - an extracellular matrix protein involved in cell migration), are not required for cell reprogramming yet are necessary for tissue growth/elongation. These separate processes, and their distinct regulatory networks, provide critical insights into the regenerative process and could provide differential targets for harnessing de novo tissue regeneration therapeutically.

## Results

### Differentially expressed genes during early muscle regeneration

Adult zebrafish EOMs can regenerate de novo using residual myocytes that reprogram into dedifferentiated myoblasts capable of proliferation. Starting with “post-mitotic” myocytes, the entire reprogramming process takes just under 20 h, at which time myonuclear proliferation can be detected [8]. To characterize the reprogramming process at the transcriptional level, we chose to focus on two time points – 9 and 18 hpi – that occur prior to cell cycle reentry by dedifferentiated myoblasts. RNA was isolated from uninjured lateral rectus (LR) muscles (CON) and injured LR muscles at 9 (H9) and 18 (H18) hpi (Additional file 1: Figure S18), followed by ribosomal RNA depletion and reverse transcription to prepare a cDNA library for deep sequencing utilizing an Illumina Hi-Seq platform. Four replicates were used for CON and five replicates were used for both H9 and H18; biological replicates consisted of pooled LR tissue from 15 to 20 zebrafish (sample read counts are shown in Additional file 2: Table S1). Mapping against the zebrafish reference genome sequence revealed alignment to 31,014 unique features.

**Fig. 1:**
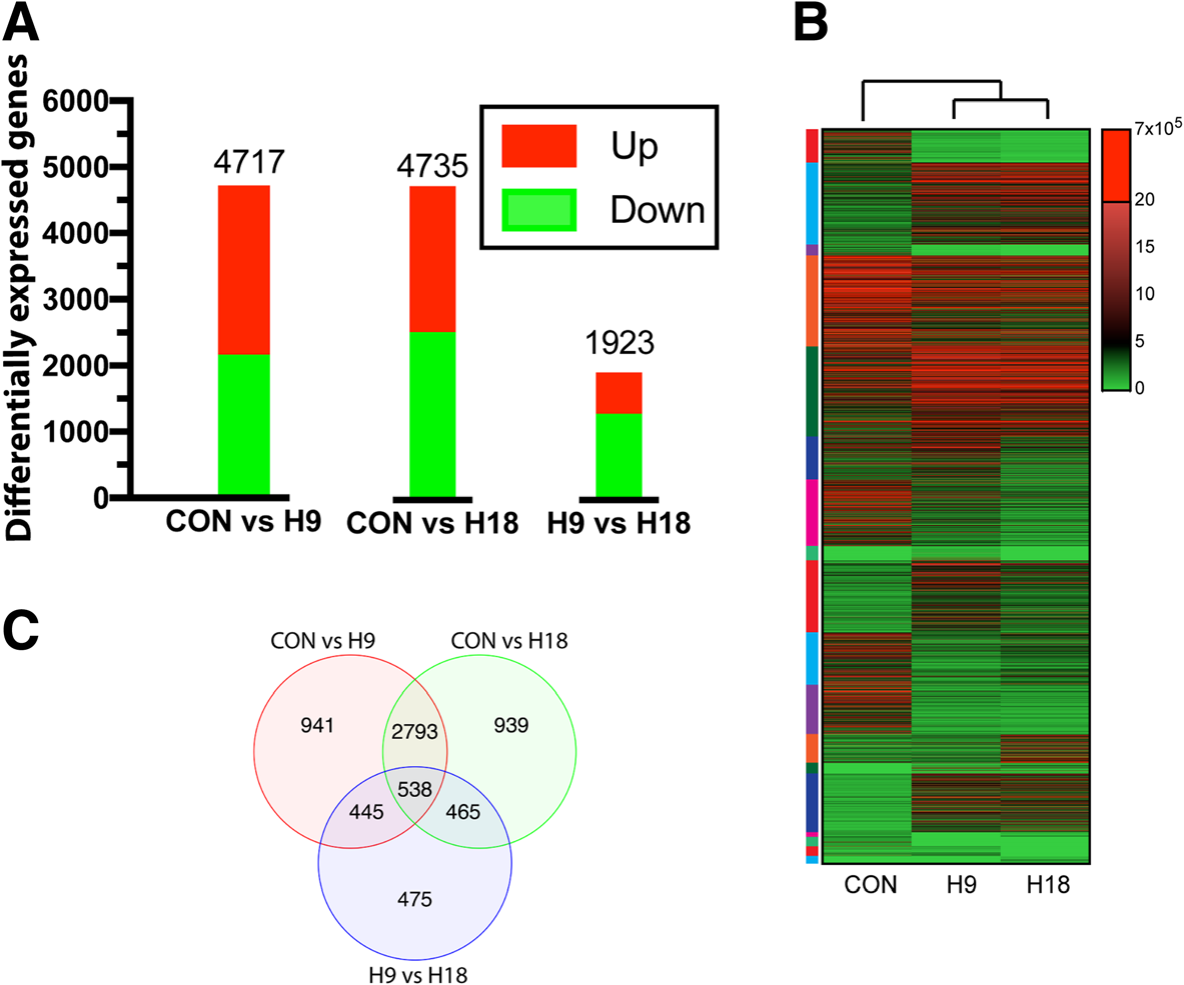
Differential gene expression during muscle regeneration. **a** Distribution of DEG of the three pair-wise comparisons. **b** Heat map of DEG. Sample names are listed below the heat maps and the expression-based hierarchical clustering separating CON from H9 and H18 samples is shown above it. Color scale (right) indicates gene expression (FPKM). FPKM <5 is shown in green and indicates low transcript abundance. FPKM >5 is shown in red and indicates high transcript abundance. DEG are ordered according to numeric clusters from Fig. 4, represented as a color bar on the left side (color code is shown in Fig. 4 over each cluster plot), listed in Additional file 3: Table S2, and Additional file 26: Figure S14 shows an enlarged heat map with the genes symbols on the left. **c** Venn diagram showing overlap of DEG between CON, H9, and H18 sample comparisons.

A total of 6596 unique differentially expressed genes (DEG) were identified among CON, H9, and H18 samples. There were more DEGs in CON vs. H9 and CON vs. H18 comparisons (4717 and 4735 genes, respectively) than in H9 vs. H18 comparisons (1923 genes) (Fig. 1a). A heat map of all DEG revealed the occurrence of multiple gene subsets with distinct expression patterns (Fig. 1b). 538 DEGs were common to all three comparisons and the highest overlap occurred between CON vs. H9 and CON vs. H18 comparisons (2793 genes) (Fig. 1c). Gene names, fold changes, and FDR values for all DEGs are included in Additional file 3: Table S2.

### Functional classification of differentially expressed genes (DEG)

**Fig. 2:**
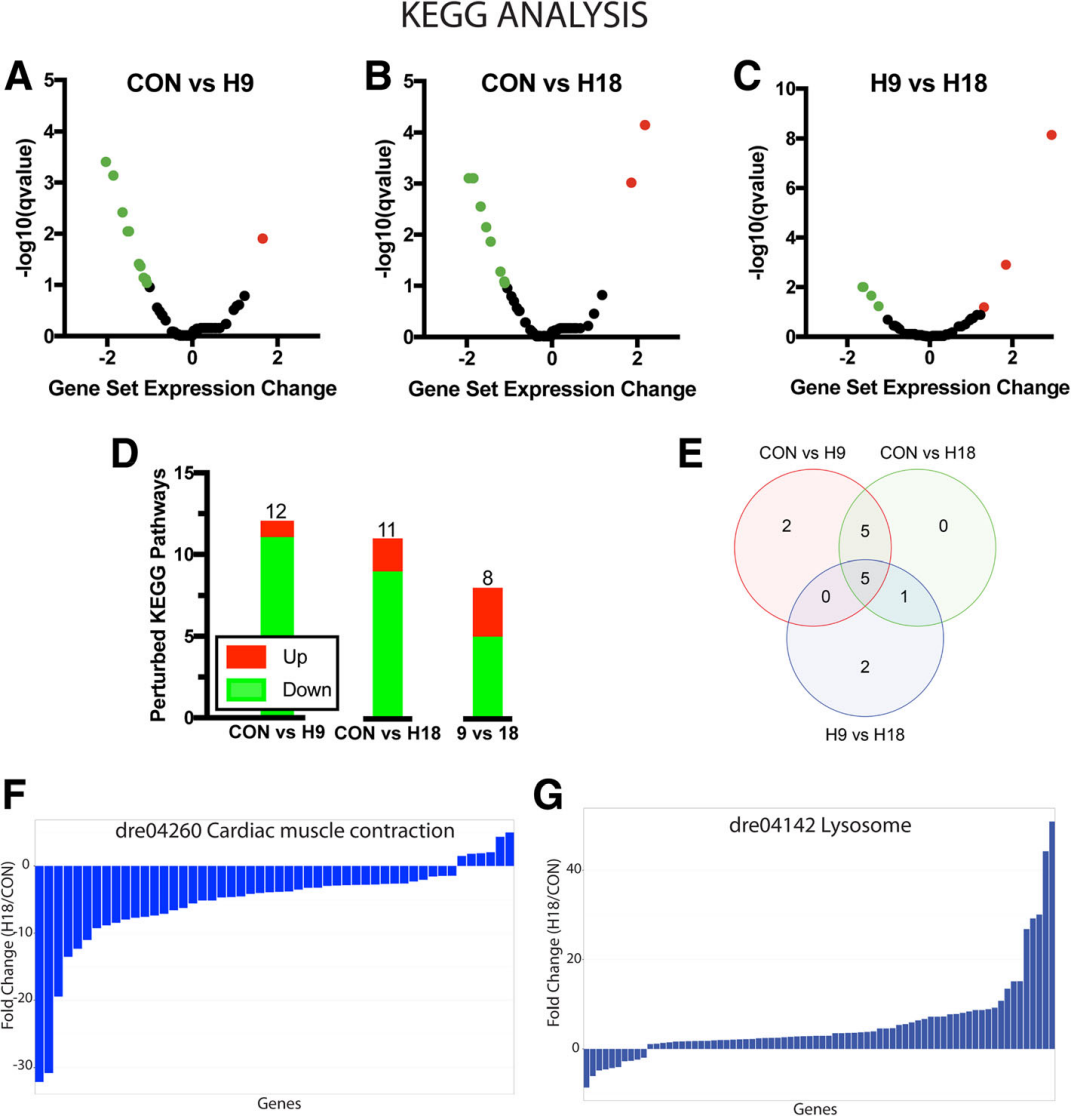
KEGG pathway classification of differentially expressed genes. Volcano plots of KEGG pathways in CON vs H9 (**a**), CON vs H18 (**b**), and H9 vs. H18 (**c**) comparisons. Gene set expression change (x-axis) of DEG within a KEGG pathway is a normalized enrichment score based on comparisons between different time points and relative to the entire DEG gene set. Significantly downregulated pathways are shown in green and significantly upregulated pathways are shown in red. **d** Distribution of significantly perturbed KEGG pathways in the three pair-wise comparisons. Significantly downregulated terms are shown in green. Significantly upregulated terms are shown in red. Cutoff for significance was q < 0.1 according to default values and parameters of the R package “gage” v2.22.0. **e** Venn diagram showing overlap of perturbed KEGG terms between CON, H9, and H18 sample comparisons. Cutoff for significance was q < 0.1 according to default values and parameters of the R package “gage” v2.22.0. Fold change (H18/CON) of the DEG of the dre04260 Cardiac muscle contraction (**f**) and dre04142 Lysosome (**g**) KEGG pathways

Comparisons between CON, H9, and H18 samples revealed that the majority of significantly perturbed KEGG pathways were downregulated (Fig. 2a-e; Additional file 4: Table S3). About half of these downregulated pathways (5 of 11) were perturbed in CON vs. H9 and CON vs. H18 comparisons but not in the H9 vs. H18 comparison, thus indicating a rapid and sustained change starting at 9 hpi (Additional file 4: Table S3); see cardiac muscle contraction pathway (dre04260) for example (Fig. 2f; Additional file 5: Figure S1A-C, Additional file 6: Figure S2). Almost all other downregulated pathways (4 of 11) were perturbed in each comparison (Additional file 4: Table S3) indicating a gradual change over time; see calcium signaling pathway (dre04020) for example (Additional file 7: Figure S3). On the other hand, upregulation of the phagosome pathway (dre04145) was common to all three comparisons (Table S3) while the lysosome pathway (dre04142) was upregulated in only CON vs. H18 and H9 vs. H18 comparisons (Fig. 2g; Additional file 5: Figure S1D-F, Additional file 8: Figure S4).

The magnitude of lysosome pathway (dre04142) upregulation between H9 and H18 was the largest of all comparisons suggesting a critical role during muscle remodeling at 18 hpi. The downregulation of the pathways for pyruvate metabolism (dre00620; Additional file 9: Figure S5), oxidative phosphorylation (dre00190; Additional file 10: Figure S6), citrate cycle (TCA cycle) (dre00020; Additional file 11: Figure S7), 2-oxocarboxylic acid metabolism (mainly TCA cycle components; dre01210; Additional file 12: Figure S8), glycolysis/gluconeogenesis (dre00010; Additional file 13: Figure S9), and carbon metabolism (dre01200; Additional file 14: Figure S10) suggests a reduction in the mitochondrial oxidative capacity of reprogramming cells. Interestingly, genes of the MAPK signaling pathway (dre04010; Additional file 15: Figure S11) showed a very dynamic expression pattern (Additional file 16: Figure S12) that was only significantly downregulated between H9 and H18 (Additional file 4: Table S3). This would indicate a change in the cellular MAPK configuration during reprogramming and expands on our characterization of the Erk pathway in EOM regeneration [19].

**Fig. 3:**
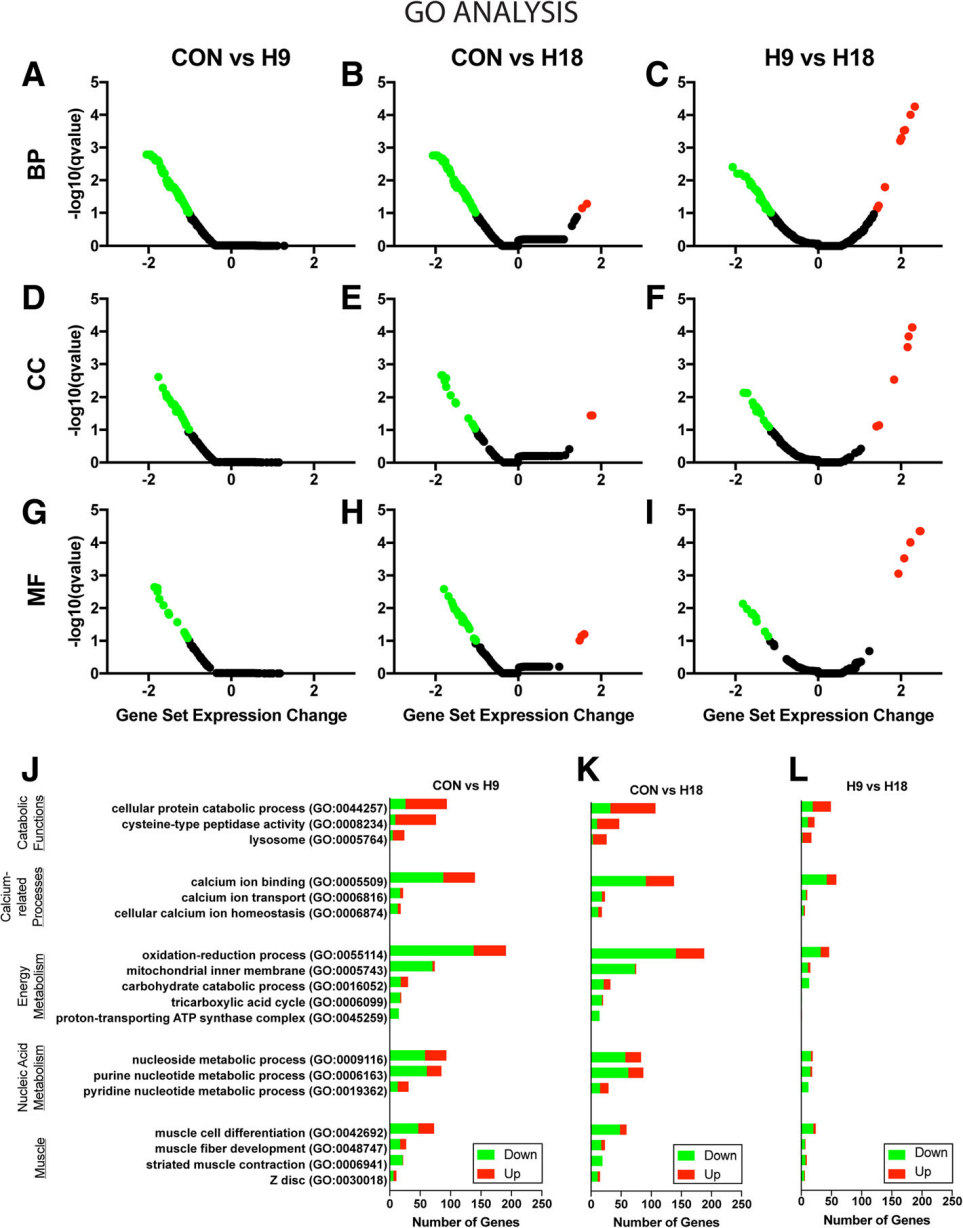
GO enrichment analysis of differentially expressed genes. Volcano plots of significantly perturbed GO terms of biological process (BP; **a**, **b**, **c**), cellular component (CC; **d**, **e**, **f**) and molecular function (MF; **g**, **h**, **i**) in CON vs H9 (**a**, **d**, **g**), CON vs H18 (**b**, **e**, **h**), and H9 vs. H18 (**c**, **f**, **i**) comparisons. Gene set expression change (x-axis) of DEG mapped to GO term is a normalized enrichment score based on comparisons between different time points and relative to the entire DEG gene set. Significantly downregulated terms are shown in green and significantly upregulated terms  are shown in red. Cutoff for significance was q < 0.1 according to default values and parameters of the R package “gage” v2.22.0. Distribution of DEG of the terms in the CON vs H9 (**j**), CON vs H18 (**k**), and H9 vs. H18 (**l**) comparisons

Comparisons between CON, H9, and H18 samples revealed that, like KEGG pathways, the majority of significantly perturbed GO terms were downregulated (Fig. 3a-i; Additional file 17: Figure S13A, B; Additional file 18: Table S4). Consequently, there was a general expression decrease of genes included in these GO terms (Fig. 3k-l). GO terms downregulated in all three comparisons (representing a gradual downregulation) and in CON vs. H18 and H9 vs. H18 (representing a late decrease in gene expression levels) were related to similar categories including muscle development, differentiation and function [muscle fiber development (GO:0048747), muscle cell differentiation (GO:0042692, Additional file 17: Figure S13C), z disc (GO:0030018), and striated muscle contraction (GO:0006941)]; nucleic acid metabolism [nucleoside metabolic process (GO:0009116), pyridine nucleotide metabolic process (GO:0019362), and purine nucleotide metabolic process (GO:0006163)]; energy metabolism [oxidation-reduction process (GO:0055114), tricarboxylic acid cycle (GO:0006099), proton-transporting ATP synthase complex (GO:0045259), mitochondrial inner membrane (GO:0005743), and carbohydrate catabolic process (GO:0016052, Additional file 17: Figure S13D)]; and calcium related processes [cellular calcium ion homeostasis (GO:0006874), calcium ion binding (GO:0005509), and calcium ion transport (GO:0006816)]. Interestingly, genes of GO terms related to catabolic functions were mainly upregulated at the expression level (Fig. 3j–l). Specifically, GO term upregulation was only observed in CON vs. H18 and H9 vs. H18 comparisons and consisted primarily of catabolic functions such as cysteine-type peptidase activity (GO:0008234), lysosome (GO:0005764, Additional file 17: Figure S13E), and cellular protein catabolic process (GO:0044257). The internal consistency between the KEGG pathways and GO terms obtained (e.g. cardiac muscle contraction and striated muscle contraction, calcium signaling pathway, cellular calcium ion homeostasis, and lysosome in both KEGG and GO analyses) supports the validity of the results.

### Gene expression profiles

**Fig. 4:**
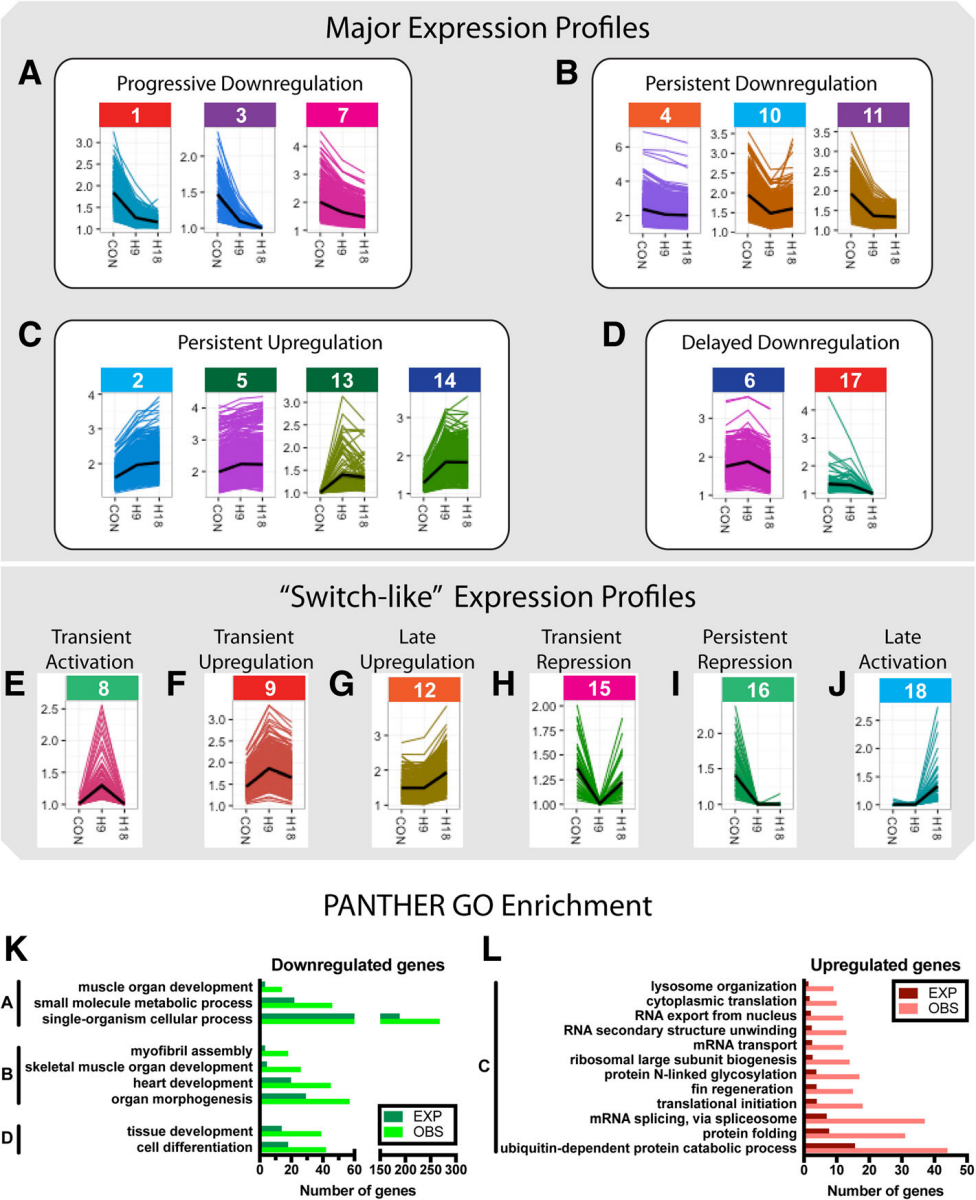
Expression profiles of gene clusters. K-means method was used to divide DEG into 18 clusters that were grouped, according to their expression profiles, into 4 major profiles (**a-d**) and 6 profiles composed of single gene clusters (**e-j**). **a** The first major profile, “Progressive Downregulation” (clusters 1, 3, and 7) showed progressive and significant downregulation at 9 hpi and 18 hpi. **b** The second major profile, “Persistent Downregulation” (clusters 4, 10, and 11) grouped downregulated genes at 9 hpi that maintained similar expression levels through 18 hpi. **c** Genes of the third major profile, “Persistent Upregulation” (clusters 2, 5, 13, and 14), were upregulated at 9 hpi and maintained through 18 hpi. **d** The fourth major profile, “Delayed Downregulation” (clusters 6 and 17) included genes whose expression was significantly downregulated only at 18 hpi. The remaining single cluster profiles (**e-j**) presented more dynamic time changes. **e** “Transient Activation” (cluster 8) contains genes that were only expressed at 9 hpi. **f** “Transient Upregulation” (cluster 9) represents genes present in control that were significantly upregulated at 9 hpi and returned to control levels at 18 hpi. **g** “Late Upregulation” (cluster 12) contains genes whose expression was upregulated at 18 hpi. **h** “Transient Repression” (cluster 15) is composed of genes expressed in control muscles and at 18 hpi but not expressed at 9 hpi. **i** “Persistent Repression” (cluster 16) contains genes that were only expressed in control muscles. **j** “Late Activation” (cluster 18) displayed the opposite trend with genes only expressed at 18 hpi. The color behind the cluster number is related to the color bar in Fig. 1 and Additional file 26: Figure S14. **k**, **l** GO enrichment analysis of the major gene expression profiles A-D. (**k**) Significantly overrepresented GO categories in profiles with genes downregulated over time (profiles A, B and D). (**l**) Significantly overrepresented GO categories in profile C which had persistently upregulated genes. Categories with *P* < 0.05 were considered as significantly overrepresented

K-means clustering of DEG identified 18 clusters that were further arranged by expression patterns into 4 major profiles composed of multiple gene clusters and 6 minor profiles composed of a single gene cluster (Fig. 4, Additional file 3: Table S2 includes the whole list of genes and its assigned cluster/profile). The first major profile, “A: Progressive Downregulation”, was composed of 994 genes (clusters 1, 3, and 7, Fig. 4a) and showed progressive and significant downregulation at 9 hpi and 18 hpi. This profile groups together genes such as myogenic factor 6 (myf6); collagen, type XV, alpha 1a (col15a1a); obscurin, cytoskeletal calmodulin and titin-interacting RhoGEF a (obscna); popeye domain containing 2 (popdc2); acetylcholinesterase (ache); matrix metallopeptidase 23bb (mmp23bb); serine peptidase inhibitor, Kunitz type 1 b (spint1b); Indian hedgehog homolog a (ihha); lefty2 (lft2); titin, tandem duplicate 1 (ttnb); periostin, osteoblast specific factor b (postnb); sarcoglycan, delta, dystrophin-associated glycoprotein (sgcd); SIX homeobox 1b (six1b); SET and MYND domain containing 1a (smyda1); and SWI/SNF related, matrix associated, actin dependent regulator of chromatin, subfamily a, member 1 (smarca1).

The second major profile, “B: Persistent Downregulation”, was composed of 1723 genes (clusters 4, 10, and 11, Fig. 4b) and showed downregulation at 9 hpi that was maintained at similar levels through 18 hpi. Representative genes of this profile include laminin alpha 2 (lama2); myogenin (myog); collagen, type VI, alpha 1 (col6a1); caveolae associated protein 4a (murca); myogenic differentiation 1 (myod1); myocyte enhancer factor 2d (mef2d); insulin-like growth factor 1a receptor (igf1ra); sarcoglycan beta, dystrophin-associated glycoprotein (sgcb); myosin, heavy polypeptide 2, fast muscle specific (myhz2); laminin beta 2, laminin S (lamb2); dystropin (dmd); tropomodulin 4, muscle (tmod4); tropomodulin 1 (tmod1); NDRG family member 4 (ndrg4); insulin-like growth factor binding protein 3 (igfbp3); LIM domain binding 3a (ldb3a); and RNA binding motif protein 24b (rbm24b).

The third major profile, “C: Persistent Upregulation”, was composed of 2172 genes (clusters 2, 5, 13, and 14, Fig. 4c) that were upregulated at 9 hpi and maintained their expression levels through 18 hpi. Representative examples from this profile include DNA (cytosine-5-)-methyltransferase 3 alpha a (dnmt3aa); SWI/SNF related, matrix associated, actin dependent regulator of chromatin, subfamily a, member 5 (smarca5); ATPase, H+ transporting, lysosomal, V1 subunit E1b (atp6v1e1b); RNA binding protein, fox-1 homolog (*C. elegans*) 2 (rbfox2); selenoprotein N, 1 (sepn1); protein arginine methyltransferase 1 (prmt1); metastasis associated 1 family, member 2 (mta2); histone deacetylase 1 (hdac1); proteasome 26S subunit, non-ATPase 11b (psmd11b); ubiquitin-like modifier activating enzyme 1 (uba1); mesoderm posterior aa (mespaa); cardiotrophin-like cytokine factor 1 (clcf1); minichromosome maintenance 10 replication initiation factor (mcm10); integrin, alpha 6a (itga6a); myelocytomatosis oncogene homolog (myc h); SWI/SNF related, matrix associated, actin dependent regulator of chromatin, subfamily a, member 4a (smarca4a); matrix metallopeptidase 9 (mmp9); and regulator of chromosome condensation 2 (rcc2).

The fourth major profile, “D: Delayed Downregulation”, was composed of 477 genes (clusters 6 and 17, Fig. 4d) that were unchanged through 9 hpi but were significantly downregulated at 18 hpi. The following are representative examples of the genes included in this profile: autophagy/beclin-1 regulator 1a (ambra1); unc-45 myosin chaperone B (unc45b); cadherin 5 (cdh5); insulin-like growth factor 1b receptor (igf1rb); actin, alpha 2, smooth muscle, aorta (acta2); methionine adenosyltransferase II, alpha a (mat2aa); and troponin I type 1a, skeletal, slow (tnn1ai).

The remaining single cluster profiles represented more dynamic “switch-like” expression patterns (Fig. 4e-j) and, as such, were of particular interest. Of those, “E: Transient Activation” (cluster 8, Fig. 4e) contained 129 genes (such as mesoderm posterior bb, mespbb; nanor, nnr; hemoglobin beta embryonic-2, hbbe2; tetraspanin 34, tspan34; and claudin 1, cldn1) that were only expressed at 9 hpi, while “F: Transient Upregulation” (cluster 9, Fig. 4f) contained 648 genes (e.g. enhancer of zeste 2 polycomb repressive complex 2 subunit, ezh2; coactivator-associated arginine methyltransferase 1, carm1; SWI/SNF related, matrix associated, actin dependent regulator of chromatin, subfamily d, member 1, smarcd1; protein arginine methyltransferase 5 and 7, prmt5 and prmt7; MYC proto-oncogene, bHLH transcription factor b, mycb) that were present in uninjured muscles (CON), peaked at 9 hpi, and returned to control levels at 18 hpi. Such rapid yet transient changes in expression suggest involvement of these gene clusters in the initiation of cell reprogramming. Profile “G: Late Upregulation” (cluster 12, Fig. 4g) contained genes upregulated at 18 hpi such as fibronectin 1a (fn1a); DNA (cytosine-5-)-methyltransferase 1 (dnmt1); midkine a (mdka); DNA primase subunit 2 (prim2); or DNA topoisomerase II alpha (top2a). Profile “H: Transient Repression” (cluster 15, Fig. 4h) contained 42 genes present in uninjured (CON) muscles and at 18 hpi but not expressed at 9 hpi. Representative examples are histone H2A, sperm-like; claudin d (cldnd); period circadian clock 1a (per1a); and glutathione peroxidase 7 (gpx7). Profile “I: Persistent Repression” (cluster 16, Fig. 4i) contained 85 genes that were only expressed in uninjured (CON) muscles (e.g. chymotrypsinogen B1, ctrb1; cysteine three histidine 1, cth1; cyclin A1, ccna1) while the 67 genes of profile “J: Late Activation” (cluster 18, Fig. 4j) displayed the opposite trend and were only expressed at 18 hpi (although this was the cluster with the highest content of non-coding transcripts, see below, some representative coding genes are aspartic peptidase, retroviral-like 1, asprv1; chemokine (C-C motif) ligand 27b, ccl27b; and heparan sulfate 6-O-sulfotransferase 3a, hs6st3a).

Panther GO term analysis of the four major profiles revealed GO terms consistent with the previous analysis conferring reliability to the results (Fig. 4k, l). Analysis of “A: Progressive Downregulation” genes showed enrichment in categories of muscle organ development (GO:0007517), small molecule metabolic process (GO:0044281), and single-organism cellular process (GO:0044763) (Fig. 4k, Additional file 19: Table S5). Panther analysis identified 15 different enriched categories in “B: Persistent Downregulation” (Fig. 4k, Additional file 20: Table S6), including skeletal muscle organ development (GO:0060538), myofibril assembly (GO:0030239), heart development (GO:0007507), and organ morphogenesis (GO:0009887). “C: Persistent Upregulation” had 31 overrepresented categories (Fig. 4l, Additional file 21: Table S7), the highest number of any Panther analysis. Several RNA processing [such as mRNA splicing, via spliceosome (GO:0000398), RNA secondary structure unwinding (GO:0010501), RNA export from nucleus (GO:0006405), mRNA transport (GO:0051028)] and protein translation [protein folding (GO:0006457), translational initiation (GO:0006413), ribosomal large subunit biogenesis (GO:0042273), cytoplasmic translation (GO:0002181)] or modification [protein folding (GO:0006457) and protein N-linked glycosylation (GO:0006487)] categories were identified. At the same time, protein degradation [ubiquitin-dependent protein catabolic process (GO:0006511) and lysosome organization (GO:0007040)] and fin regeneration (GO:0031101) categories were also enriched. Enriched categories in “D: Delayed Downregulation” (Fig. 4k, Additional file 22: Table S8) were related to developmental and differentiation processes such as cell differentiation (GO:0030154) or tissue development (GO:0009888).

Interestingly, the Panther GO analysis of single cluster profiles revealed relevant terms not noted in other profiles or the GAGE analysis. Enriched terms in “F: Transient Upregulation” (Additional file 23: Table S9) were related to the categories of gene transcription and ribosome formation and included: regulation of gene expression, epigenetic (GO:0040029), transcription, DNA-templated (GO:0006351), and maturation of SSU-rRNA from tricistronic rRNA transcript (SSU-rRNA, 5.8S rRNA, LSU-rRNA) (GO:0000462). Enriched categories in “G: Late Upregulation” (Additional file 24: Table S10) were related to DNA replication and repair [DNA replication initiation (GO:0006270), mismatch repair (GO:0006298), and mitotic chromosome condensation (GO:0007076)]. Profiles “E: Transient Activation”, “H: Transient Repression”, “I: Persistent Repression”, and “J: Late Activation” did not have enriched GO terms or categories because of the low percentage of GO annotations for genes in these clusters (Table 1). Interestingly, the gene clusters with lowest percentage of GO annotated terms had both a higher percentage of lncRNAs (Table 2), which remain to be functionally annotated, and the most dynamic “switch-like” expression patterns (Fig. 4).

**Table 1:** GO term annotations for gene expression profiles

Profile (cluster)	Gene number	Annotated genes	%
A (1/3/7)	994	671	67.51
B (4/10/11)	1723	1296	75.22
C (2/5/13/14)	2172	1682	77.44
D (6/17)	477	309	64.78
E (8)	129	23	17.83
F (9)	648	476	73.46
G (12)	259	32	12.36
H (15)	42	14	33.33
I (16)	85	24	28.24
J (18)	67	11	16.42

**Table 2:** Distribution of coding (cRNA) and long non-coding (lncRNA) RNA by cluster

Cluster	Total	cRNA (%)	IncRNA (%)
1	301	97.67	2.33
2	738	99.86	0.14
3	98	83.67	16.33
4	813	98.65	1.35
5	810	99.38	0.62
6	389	98.71	1.29
7	595	98.32	1.68
8	129	71.32	28.68
9	648	99.23	0.77
10	472	99.36	0.64
11	438	98.86	1.14
12	259	100.00	0.00
13	96	82.29	17.71
14	528	99.43	0.57
15	42	73.81	26.19
16	85	77.65	22.35
17	88	73.86	26.14
18	67	62.69	37.31

### Expression timing correlates with temporal roles during muscle regeneration

**Fig. 5:**
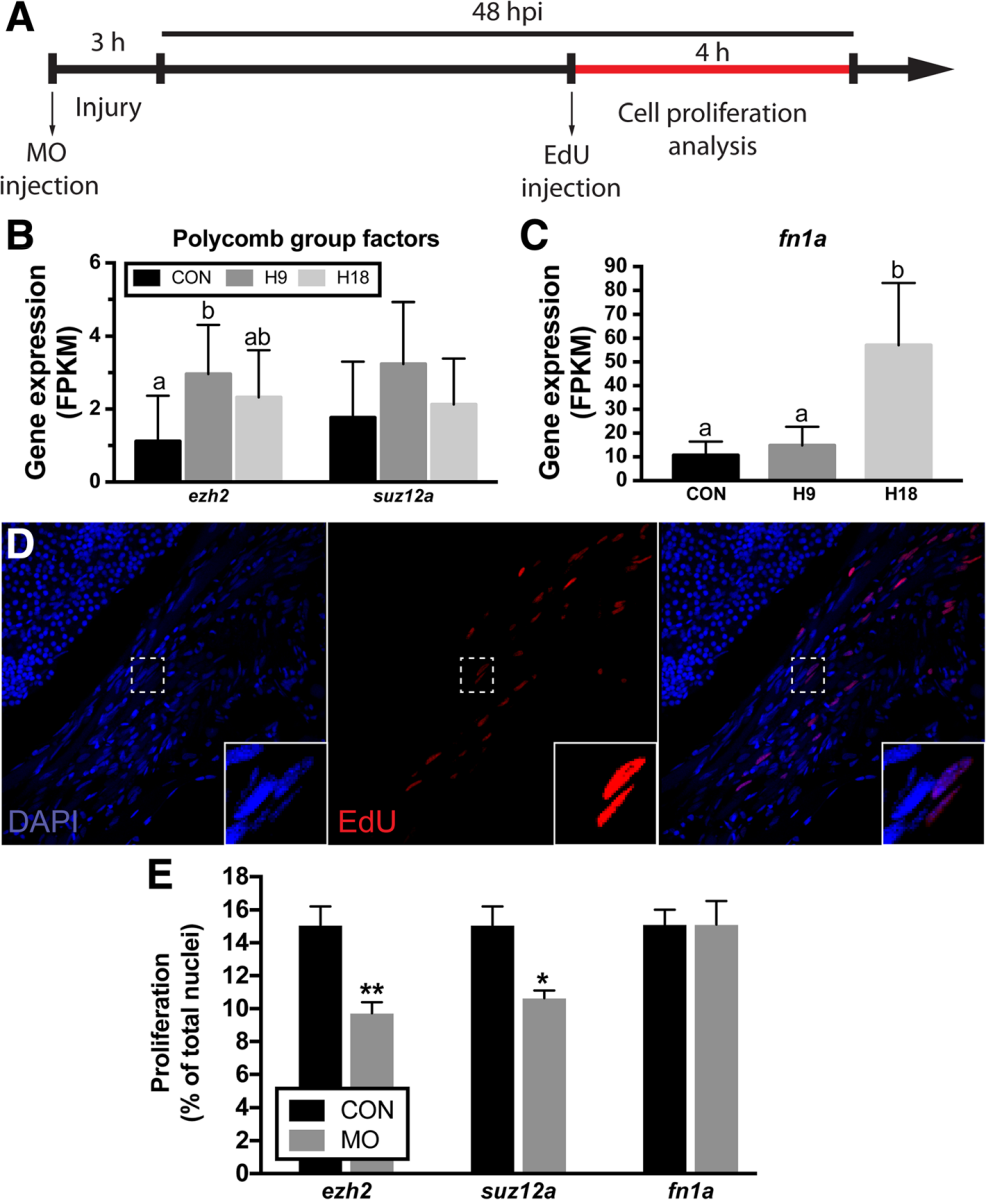
Proliferation following knockdown of select differentially expressed genes. **a** LR regeneration involves a proliferative burst that generates enough cells to replace lost tissue. EdU assays were performed according to the schematic. **b** Gene expression (FPKM) of epigenetics selected genes, *ezh2* and *suz12a*. **c** *Fibronectin 1a* gene expression (FPKM). **d** Confocal microscopy of cell proliferation in the regenerating muscle. Inset shows higher resolution detail of the box in the panel. DAPI staining (blue) shows the total number of nuclei in the muscle (left) and EdU staining (red) shows proliferating nuclei (middle). Merged panel (right). **e** Quantification of cell proliferation in injured LR at 48 hpi. Values are averages ± SEM (*n* ≥ 5) in control MO or target gene MO injected fish. Different letters (a, b, ab) in (**b**) and (**c**) indicate significant differences among time points. * *P* < 0.05; ** *P* < 0.01; Student’s t-test

**Fig. 6:**
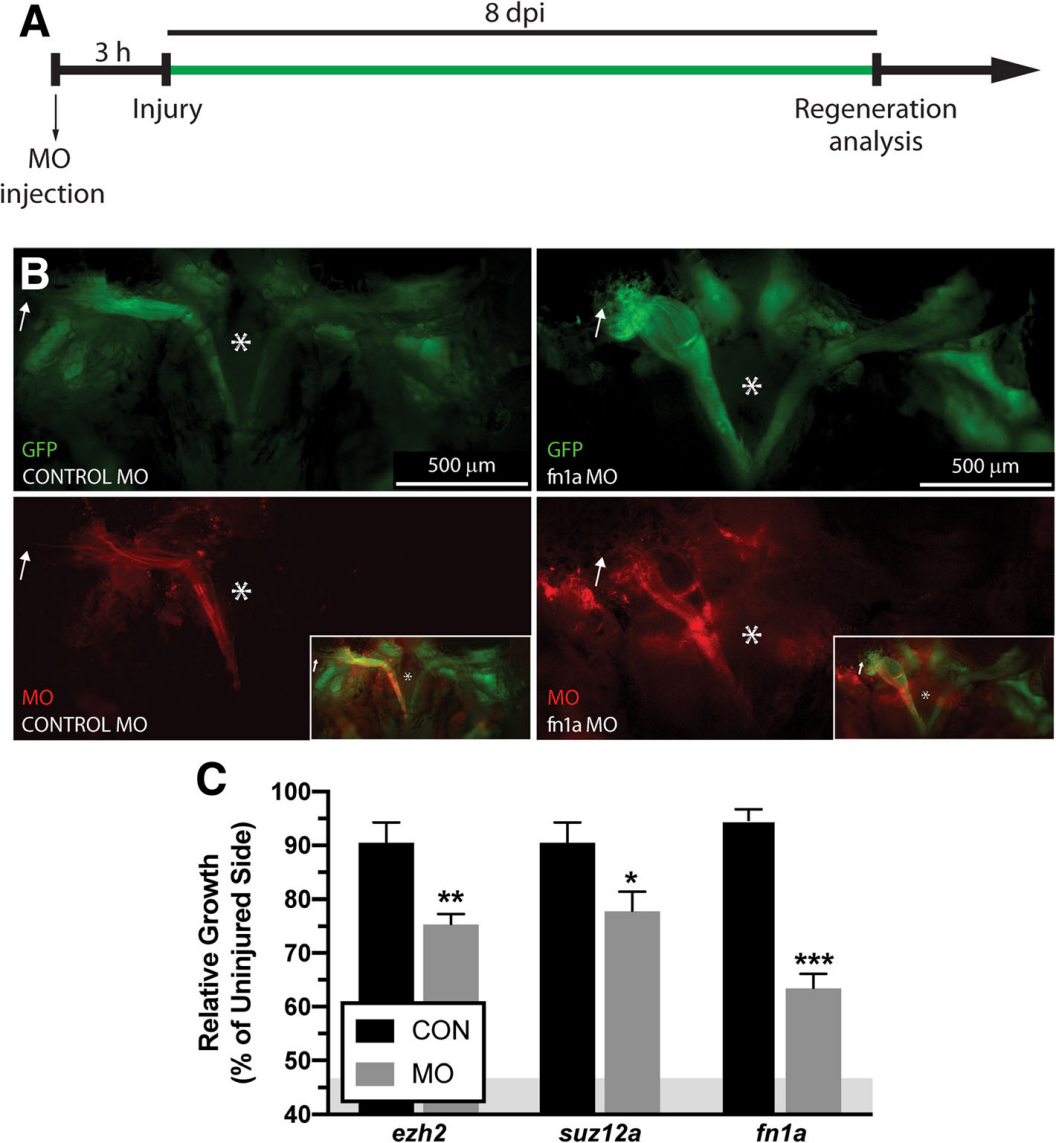
Regeneration following knockdown of select differentially expressed genes. **a** LR regeneration assays were performed according to the schematic. **b** Craniectomy was performed to visualize EGFP-labeled muscle at 8 dpi. Brain was removed to allow visualization of the skull base (*) Microinjected MOs are detected throughout the entire regenerating muscle, including the distal ends (white arrow). Control MO (left) and fn1a MO (right) injected fish are pictured, Additional file 26: Figure S14 shows representative examples of injected fish with MO targeting the rest of the genes. **c** Quantification of LR regeneration at 8 dpi, all MOs targeting specific mRNA decreased muscle regeneration at 8 dpi compared to control MO injected fish. Values are averages ± SEM (*n* = 5–7) in control MO or target gene MO injected fish. *P < 0.05; **P < 0.01; *** *P* < 0.001; Student’s t-test. The residual muscle left following myectomy surgery (46.77 ± 4.8%, average ± SD) is represented as a grey area in C

As anticipated, a large subset of epigenetic regulators and transcription factors were induced early in the reprogramming process (profile “F: Transient Upregulation”; Fig. 4f). In fact, the GO term “regulation of gene expression, epigenetic” (GO:0040029) was significantly enriched in profile F. All this suggested that at least a subset of these factors might be mechanistically involved in allowing post-mitotic myocytes to dedifferentiate into proliferative myoblasts and we thus decided to focus on transcription factors and epigenetic regulators. We further hypothesized that early-activated genes would play a particularly important role in the reprogramming and dedifferentiation of injured myocytes, and tested the hypothesis using gene knockdown experiments using antisense morpholino oligonucleotide (MO), a technique widely used to perform tissue-specific knockdown experiments in adult zebrafish [20–22] and other model organisms such as adult axolotls [23], xenopus larvae [24] or chick [25] and mouse [26] embryos. Briefly, lissamine-tagged MOs are injected and then electroporated into the LR muscle 3 h prior to myectomy injury (Figs. 5a and 6a). LR-specific MO uptake is detected via lissamine fluorescence within muscle fibers, persisting through 8 dpi, and without affecting the adjoining EOM (Fig. 6b, Additional file 25: Figure S15). Proliferation at 48 hpi is assayed via EdU uptake as a surrogate for myocyte reprogramming, since proliferation of dedifferentiated myoblasts represents the final step of the reprogramming process (Fig. 5e). Additionally, we physically measure the length of the regenerating LR muscle in order to assess anatomic regeneration (Fig. 6b).

Because of their rapid induction (Fig. 5b), gene induction in a “switch-like” fashion (i.e. expression profile “F: Transient Upregulation”), and known roles as epigenetic regulators of cell identity [27], we assessed the role of Polycomb group factors in myocyte reprogramming. Knockdown of the Polycomb Repressive Complex 2 (PRC2) factors Ezh2 and Suz12a revealed a significant reduction in the number of proliferating dedifferentiated myoblasts during what is normally peak proliferation [8], between 44 and 48 hpi (Fig. 5e), supporting a key role in the reprogramming process. Furthermore, physical measurement of LR muscle length following Polycomb group factor knockdown revealed a significant delay in muscle regeneration (Fig. 6c, Additional file 26: Figure S14 D-I) that was consistent with the inhibition of reprogramming and subsequent proliferation. These findings reveal a key role for PRC2 factors in regulating myocyte reprogramming.

Next, we hypothesized that since myocyte dedifferentiation to myoblasts represents the first step of the muscle-to-mesenchymal transition (MMT) observed during EOM regeneration [8], later activated (i.e. 18 hpi) genes would not be related to myocyte reprogramming and cell cycle reentry but rather play important roles in muscle tissue anatomic regeneration. Among genes in the single cluster profile “G: Late Upregulation”, *fn1a*, encoding fibronectin – an extracellular matrix (ECM) glycoprotein whose expression is activated during mesenchymal transitions and promotes cell migration [28] – was notable due to its magnitude of induction between 9 and 18 hpi (Fig. 5c). As hypothesized, knockdown of Fn1a had no effect on cell proliferation (Fig. 5e). However, it resulted in a large reduction in anatomic LR muscle tissue regeneration (Fig. 6b and c). This is consistent with the hypothesis that genes activated later in the response were: (1) not significant for the cellular reprogramming process, and (2) more important for later events of the regenerative process that include muscle growth through myoblast migration and/or myofiber fusion.

## Discussion

Differentiated cells are marked by an epigenetic program that determines which genes are to be expressed or repressed in a genome-wide fashion [29–31]. The myocyte program involves expression of genes encoding sarcomeric proteins and repression of cell cycle genes and those associated with different, non-muscle tissue types (e.g. liver). Reprogramming a differentiated cell into a different identity, whether or not of similar cell lineage, would involve reprogramming every aspect of that cell’s biology – its transcriptome, metabolome, and proteome. Myocytes are among the most specialized of cells and are considered “post-mitotic.” Yet, in zebrafish EOMs, catastrophic muscle injury triggers rapid myocyte reprogramming (within 20 hpi) resulting in a large population of proliferating dedifferentiated myoblasts that repopulate and de novo regenerate the absent muscle [8, 19, 32]. This surprising discovery has provided a unique opportunity to study limited cellular reprogramming that maintains lineage restriction yet is capable of regenerating skeletal muscle de novo.

In order to understand the mechanism of myocyte reprogramming, we utilized a deep sequencing approach to characterize the early transcriptional changes that occur as post-mitotic myocytes reprogram into dedifferentiated myoblasts capable of proliferation. Two parallel strategies, GAGE analysis (KEGG pathways and GO terms) or K-means clustering followed by Panther GO term enrichment analysis, were used to functionally annotate all 6596 DEGs (Additional file 5: Figure S1). Broadly speaking, both strategies gave similar results, conferring reliability to our analyses. Our results reveal that myocyte dedifferentiation is marked by downregulation of muscle-specific programs, such as the sarcomeric apparatus and calcium homeostasis. Additionally, both parallel GO term analyses (GAGE and Panther enrichment analysis) revealed a downregulation of terms more broadly related to cellular differentiation and tissue or organ morphogenesis. The downregulation of pyruvate metabolism, oxidative phosphorylation, TCA cycle pathways, pyruvate metabolic processes, proton-transporting ATP synthase complexes, and mitochondrial ATP synthesis reflects a decrease in the need for mitochondrial oxidative capacity in dedifferentiating cells. This is similar to the processes of somatic and oncogenic cellular reprogramming to a pluripotent state in which reprogrammed cells undergo metabolic “rewiring” that reduces both mitochondrial content and oxidative phosphorylation capacity [33–37]. Interestingly, autophagy activation reduces mitochondrial content early in reprogramming [38] and, although our analysis did not find any significant autophagy-specific KEGG pathways or GO terms, we have previously shown that autophagy plays a key role in zebrafish muscle regeneration [32]. The absence of significant autophagy-specific KEGG pathways or GO terms is not surprising since, in this model, autophagy is regulated mainly at the protein level [32]. Notably, we found a consistent upregulation of terms related to lysosomal protein degradation – the last step of the autophagy process – including the lysosome KEGG pathway and the GO terms cysteine-type peptidase activity and lysosome organization.

In addition to the already discussed pathways and terms, enrichment analysis of unbiased k-means clustering expression profiles revealed several interesting GO terms. Enriched GO terms within “G: Late Upregulation” genes included DNA replication, chromosome condensation, and DNA biosynthesis, all of which are consistent with reprogramming cells preparing to reenter the cell cycle. The timing of upregulation within this profile (i.e. 18 hpi) correlates well with our published results showing that cell proliferation (EdU incorporation into DNA) begins by 20–24 hpi [8]. A recent bioinformatic analysis of zebrafish heart regeneration identified similar changes in energy metabolism, amino-acid biosynthesis and DNA replication linked to the initial proliferative response [39], indicating shared molecular mechanisms between these regenerative processes. The reacquisition of proliferative potential, importantly, represents the most fundamental aspect of the reprogramming process. Supporting this finding, pharmacological inhibition of the cell cycle with either bortozemid or 5-fluorouracil (Additional file 27: Figure S16) blocks the muscle-to-mesenchymal transition that drives EOM regeneration [8].

Terms related to mRNA processing, mRNA transport, and protein translation and modification were enriched in “C: Persistent Upregulation” genes (Fig. 4c) which were upregulated at 9 and 18 hpi. Additionally, genes only upregulated at 9 hpi (“F: Transient Upregulation”, Fig. 4f) were related to transcription, rRNA maturation, tRNA processing, and epigenetic regulation of transcription. These findings indicate that the cells are preparing to synthesize a new set of proteins that, in conjunction with broad protein degradation, would allow the cell to perform the drastic cellular proteome changes that reprogramming requires [40]. Interestingly, the GO term “fin regeneration,” enriched in “C: Persistent Upregulation” genes (Fig. 4c), was upregulated at 9 and 18 hpi, and included *hdac1* (histone deacetylase 1) and *smarca4a* (SWI/SNF related, matrix associated, actin dependent regulator of chromatin, subfamily a, member 4a). These genes, along with GO terms related to epigenetic regulation of transcription, confirm the importance of chromatin-dependent gene expression changes during the reprogramming process.

Based upon functional annotations, we hypothesized that important initiators of dedifferentiation would belong to expression profile “F: Transient Upregulation”, which peak at 9 hpi and return to control levels at 18 hpi, and that morpholino knockdown of these genes would both interfere with cell cycle reentry and impair the anatomic EOM regeneration. Profile “F: Transient Upregulation” candidates were *ezh2*, encoder of Polycomb group protein that broadly regulate epigenetic states [27]. Although delayed anatomic regeneration following Ezh2 knockdown supported the regulatory importance of profile “F: Transient Upregulation” genes, the true test remained the ability to affect cell cycle reentry within dedifferentiated myoblasts. Knockdowns of Ezh2 reduced the proportion of proliferating myoblasts, thus bolstering the importance of these and other dynamically expressed profile “F: Transient Upregulation” genes as “switch”-like regulators of EOM dedifferentiation leading to regeneration. In line with our results, mammalian EZH2 promotes proliferation by modifying chromatin conformation in models of pancreas [41], liver [42], and dental pulp regeneration [43]. Importantly, knockdown of the Ezh2 Polycomb partner Suz12a replicated Ezh2 knockdown results, thus confirming the relevance of epigenetic regulation in myocyte reprogramming. In addition to the discussed role in promoting cell cycle reentry, PCR2 factors may play an additional transient role repressing muscle identity since PRC2 factors maintain the chromatin state of muscle genes in a repressive conformation and must be degraded to allow myogenic differentiation [44].

We further hypothesized that genes with later activation (18 hpi) would affect post-reprogramming processes. Profile “G: Late Upregulation” included the ECM factor *fn1a* (fibronectin) whose knockdown, unlike those in profile “F: Transient Upregulation”, resulted in a robust defect in anatomic regeneration with no effect on proliferation. Our results are supported by reports showing that *fn1a* was required for zebrafish heart regeneration but not for cardiomyocyte dedifferentiation and subsequent proliferation [45]. It was also found to be upregulated in microarray analyses of zebrafish heart [39, 46] and fin [47] regeneration, validating this transcriptomic assessment and suggesting the existence of common features among tissue regeneration models. Interestingly, *fn1a* was also identified in a correlation of fin regeneration genes with melanoma markers [48], highlighting again the similarities between regeneration and cancer. The data supports the hypothesis that while the regeneration process may take days, the transcriptional template for the entire process is determined at the outset of the regeneration process: early activated genes regulate initial reprogramming events, while late response genes regulate tissue growth. These findings reveal the temporal relationship that correlates transcriptional regulation with biological function.

## Conclusion

**Fig. 7:**
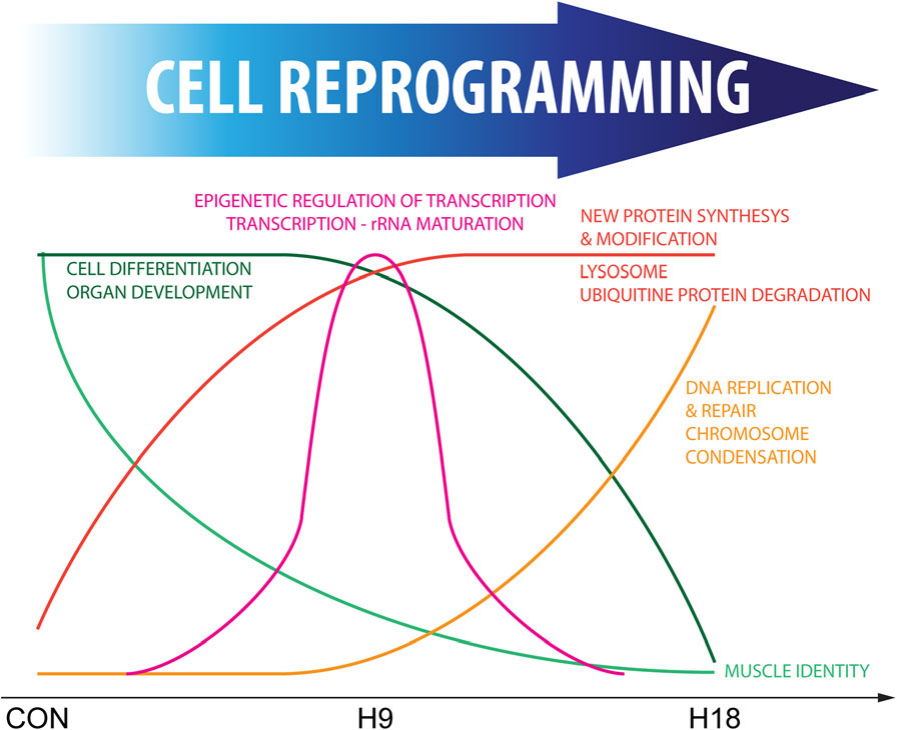
Proposed coordinated process of myocyte reprogramming. Reprogramming “post-mitotic” myocytes into dedifferentiated myoblasts would require a complex, temporally sensitive, orchestration of genes and pathways involved in the regulation of chromatin modifications, protein degradation, RNA processing, and DNA and protein synthesis.

We describe a transcriptome analysis of an in vivo dedifferentiation process during which myocytes reprogram to become myoblasts that regain the capacity to proliferate. Our analysis reveals a complex and coordinated process (Fig. 7) that begins with downregulated expression of genes that confer muscle identity, significant changes in metabolic programs, coordinated activation of protein degradation that clears the sarcomere and other muscle-specific protein complexes, and the synthesis of new proteins that reshape the proteomic cellular profile. Simultaneously, activation of programs related to DNA replication, repair, and chromosome condensation, as well as of genes required for the G-to-S transition, ultimately leads to cell cycle reentry by reprogrammed myocytes and the formation of dedifferentiated myoblasts. The early and temporally regulated activation of genes related to epigenetic regulation of transcription likely drives the broad programmatic genomic changes required for myocyte dedifferentiation. We propose a mechanistic overview of the temporal orchestration of pathways involved in cellular reprogramming. We also assess the importance of early-activated chromatin remodeling factors (Ezh2 and Suz12a) during the dedifferentiation and cell cycle reentry of “post-mitotic” myocytes. On the other hand, late activated genes, like *fn1a* (encoding an extracellular matrix interacting protein), would regulate the anatomic growth of regenerating muscle tissue following cellular reprogramming.

Understanding the molecular mechanism of cellular reprogramming that leads to lineage-restricted proliferating progenitor cells carries particular importance to the field of de novo tissue regeneration. Furthermore, the similarities between tissue regeneration and cancer (for example, cell reprogramming and dedifferentiation, proliferation and migration) may suggest a similar relationship between genes that control “stemness” and those that regulate metastasis. Future research will aim to uncover greater details of these similarities and relationships.

## Methods

### Zebrafish (*Danio rerio*) rearing and surgery

All animal work was performed in compliance with the Association for Research in Vision and Ophthalmology Statement for the Use of Animals in Ophthalmic and Vision Research and approved by the University of Michigan Committee on the Use and Care of Animals, protocol 06034. Sexually mature adult (4–18 months of age) wild-type and transgenic (α-actin::EGFP) zebrafish were spawned in our fish facility and raised according to standard protocol at 28 °C with a 14-h light:10-h dark cycle.

Adult zebrafish were anesthetized using 0.05% tricaine methanosulfate (Tricaine-S; Western Chemical, Ferndale, WA) and approximately 50% of the right lateral rectus (LR) muscle was surgically removed [8]. The amount of muscle remaining after surgery (46.77 ± 4.8%, average ± S.D.) was quantified by craniectomy as described previously [8] (Additional file 28: Figure S17 shows a diagram of zebrafish EOMs and how the regenerating muscle is measured) and is represented in the figures as a grey area. No significant mortality was noted.

### RNA isolation and sequencing

Zebrafish heads were decalcified for 2–3 days in a citrate-buffered (pH 5.6) solution containing 10% ethylenediamine-tetraacetic acid (EDTA; Thermo Fisher Scientific, Waltham, MA) saturated with ammonium sulfate (Promega, Madison, WI) to preserve RNA quality [49]. Specimens were directly placed in Shandon M-1 Embedding Matrix (Thermo Fisher Scientific, Waltham, MA) frozen on dry ice and coronal frozen sections (16 μm, cryostat temperature − 35 to −40 **°**C (CM3050S disposable blade cryostat; Leica Microsystems Inc., Buffalo Grove, IL)) were placed onto PEN-membrane framed slides (Leica, PN 11505151) and stored at −80 °C. Slides were washed and dehydrated (1 min 70% EtOH, 30 s RNAse-free water, 30 s 70% EtOH, 2 × 1 min 100% EtOH). LR muscle tissue was dissected from frozen sections using laser micro-dissection (LMD7000; Leica Microsystems Inc., Buffalo Grove, IL). Micro-dissected tissue was collected in 30 μL aliquots of RLT buffer (Qiagen, Valencia, CA) with 2-mercaptoethanol added (10 μL/mL). Pooled aliqouts from several dissected slides had areas that ranged from 27 to 71 million μm^2^ per sample. RNA was isolated in a fixed volume of 1 mL of TRIzol (Invitrogen, Inc., Carlsbad, CA) and the manufacturer’s protocol was followed except for the addition of 0.2 mL of dH2O per mL of Trizol. Phase separation after chloroform addition was performed using phase lock gel tubes - Heavy 2 mL (5-Prime, Gaithersburg, MD). The RNA was further purified on microcolumns with DNAse treatment (ReliaPrep RNA Tissue Miniprep System, Promega, Madison WI) after addition of an equal volume of 100% EtOH to the Trizol aqueous layer and application to the column. Elution was with 15 μL RNAase free water.

RNA quality and quantity were reassessed using an Agilent 2200 TapeStation (Agilent Technologies, Santa Clara, CA). RIN greater than 7 was required for all samples. Pooled purified RNA samples were used for performing ribosomal-depletion (Ribo-Zero Gold rRNA Removal Kit, Illumina) and library preparation (Illumina’s TruSeq Stranded mRNA Library Prep Kit).

Sequencing was performed by the UM DNA Sequencing Core, using an Illumina Hi-Seq (50-cycle, single end read) platform (Illumina, Inc., San Diego, CA).

### Transcriptome assembly and differentially expressed gene identification

Sequencing reads were obtained in fastq format and evaluated using FastQC v0.11.3 (http://www.bioinformatics.babraham.ac.uk/projects/fastqc/). The Tuxedo Suite was used for alignment, differential expression analysis, and post-analysis diagnostics (Additional file 1: Figure S18) [50–52]. Briefly, reads were mapped to the reference genome followed by the transcriptome (GRCz10) using TopHat v2.0.13 and Bowtie v2.2.1 with default parameters and --b2-very-sensitive, −-max-intron-length 400 (as recommended [53]), −-no-coverage-search, and --no-novel-juncs. Cufflinks/CuffDiff v2.2.1 were used for expression quantitation and differential expression analysis (NCBI GRCz10.fa, reference genome sequence; NCBI GRCz10.gtf, reference transcriptome) using parameters --multi-read-correct, −-compatible-hits-norm, and --upper-quartile –norm. Locally developed scripts were used to identify differentially expressed genes (DEG) based on three criteria: test status = “OK”, FDR < 0.05, and fold change ≥1.5 or ≤1/1.5.

### Clustering and gene set analysis

The R package “cummeRbund” v2.14.0, part of the Tuxedo Suite package, was used to perform hierarchical clustering of samples by gene expression values (csDendro function; [54]).

Kyoto Encyclopedia of Genes and Genomes (KEGG) pathway and Gene Ontology (GO) term analyses of the whole data set of DEG were performed using the R package GAGE “Generally Acceptable Gene set Enrichment” (GAGE v.2.22.0) package (Additional file 1: Figure S18) implemented in R [55, 56]. Briefly, default parameter settings were used for comparisons of log-scaled gene set expression (i.e. enrichment) data between different time points (unpaired, q-value <0.1). Gene sets were defined using annotations obtained from GAGE v2.22.0, go.db v3.2.2, and kegg.db v3.2.2. The R package “pathview” v.1.12.0 and KEGGGraph v1.30.0 were used to visualize gene set expression data in the context of functional pathways [57, 58].

CummeRbund was used to perform K-means clustering (csCluster and csClusterPlot functions) and the number of clusters (*k = 18*) was determined by evaluating k = 3 to k = 30 and the following criteria: 1) maximize the number of clusters with new expression profiles; and 2) minimize the number of clusters with similar expression profiles.

GO enrichment analysis of gene clusters (Additional file 1: Figure S18) was performed using the PANTHER (protein annotation through evolutionary relationship) classification system (http://www.pantherdb.org/) [59, 60].

### Morpholino oligonucleotide injection

Microinjection and electroporation of morpholino oligonucleotides (MOs; Gene-Tools, LLC, Philomath, OR) was used as described for knockdown experiments in adult zebrafish [20–22]. Briefly, lissamine-tagged MOs (~0.2 μL, 1 mM in nuclease-free H_2_O) were directly microinjected into the LR muscle followed by electroporation (6 to 10 pulses at 48 V/cm, BTX ECM830 electroporator; Harvard Apparatus, Holliston, MA). Microinjections were performed 3 h prior to LR injury, and MO uptake was confirmed via lissamine fluorescence prior to myectomy. MOs were designed to target the 5′-UTR of respective mRNAs (translational blocking MOs), and were compared to a standard control MO (CON) targeting a human beta-globin intron mutation. When possible, previously published MOs were utilized (Table 3). For all others, MO sequence design was performed as a service by Gene Tools. All MOs were injected at the same concentration, and all experiments were performed using 5 fish per experimental group per time point, unless stated otherwise in the text and/or figure legend. No significant mortality was noted.

**Table 3:** Sequence of the morpholino antisense oligonucleotides (MOs)

	Sequence	Reference
*ezh2*	5′-CGATTTCCTCCCGGTCAATCCCATG	[62]
*fn1a*	5′-TTTTTTCACAGGTGCGATTGAACAC	[63]
*suz12a*	5′-GAGCCATCCTAAAATAGCGTTCGTG	[64]
Control (CON)	5′-CCTCTTACCTCAGTTACAATTTATA	[65]

### Specimen processing and cell proliferation assay

Zebrafish heads were removed and fixed in 4% paraformaldehyde in PBS (PFA; Sigma-Aldrich, St. Louis, MO) overnight at 4 °C. Decalcification was performed for 48-h using Morse’s solution (45% formic acid in H_2_O, ACROS Organics, Fair Lawn, NJ; 20% sodium citrate in H_2_O, R&D Systems, Bristol, UK). Fixed and decalcified tissues were cryopreserved with 20% sucrose in PBS (ACROS Organics, Fair Lawn, NJ), embedded in OCT (Thermo Fisher Scientific, Waltham, MA), and frozen.

Cellular proliferation was assessed by intra-peritoneal injections of 5-ethynyl-2′-deoxyuridine (EdU; Invitrogen, Inc., Carlsbad, CA) [61]. Wild-type fish were injected with EdU (25 μL, 10 mM in PBS) at 44 hpi and sacrificed 4 h later (Fig. 5a). The injured muscles of 4–8 fish per experimental group were evaluated microscopically using transverse frozen sections (10 μm) as described previously [8]. EdU^+^ and total (DAPI) nuclei were counted from 3 to 4 non-sequential sections per muscle (more than 30 sections per experiment were analyzed), representing approximately 700 total nuclei (range: 296–1176) per muscle. Cell proliferation is represented as the percentage of EdU^+^ nuclei in the injured muscle of target gene MO injected fish relative to the percentage of EdU^+^ nuclei in the injured muscle of control MO injected fish.

### Regeneration assay

Transgenic α-actin::EGFP zebrafish were used to visualize the LR muscles and measure regeneration by craniectomy 8 dpi (Fig. 6a) as described previously [8]. Briefly, calvarial bones (top of the skull) and the brain were removed to allow visualization of the skull base where both LR muscles originate. Regeneration is represented as the length of the injured muscle compared to the non-injured muscle of MO injected fish relative to the length of the injured muscle compared to the non-injured muscle of control MO injected fish. All experiments were performed using 5 fish per experimental group per time point unless stated otherwise in the text and/or figure legend.

## Additional files

Additional file 1: Figure S18. Diagram of the strategy of the present study. RNA was isolated and sequenced from laser microdissected adult zebrafish LR muscle tissue at multiple time points post injury. Transcriptome assembly and DEG identification was performed using the Tuxedo Suite. DEG functional classification was concurrently performed via separate strategies using either gene set enrichment analysis (left: KEGG pathway, GO term analysis [GAGE]) or expression profile-based clustering (right: K-means clustering [cummeRbund] and GO enrichment [Panther]). MO knockdown-based phenotypic screens included both cell proliferation and overall regeneration assays as readouts of DEG functional significance. (TIFF 18214 kb)

Additional file 2: Table S1. Read counts and alignment rates. (DOCX 17 kb)

Additional file 3: Table S2. List of DEG with gene ID, symbol and name. Expression profile, K-means cluster, FPKMs, fold changes, and q values are also shown. (XLSX 1397 kb)

Additional file 4: Table S3. Significantly perturbed KEGG pathways. Table is ordered according to the composite log scaled expression of all genes assigned to a given pathway. Significantly downregulated pathways are shown in green. Significantly upregulated pathways are shown in red. Cutoff for significance was q < 0.1 according to default values and parameters of the R package “gage” v2.22.0. (XLSX 68 kb)

Additional file 5: Figure S1. Cardiac muscle contraction and lysosome KEGG pathway gene expression. Related to Fig. 2. Dot plot of gene expression (FPKM, CON vs H9, CON vs 18, H9 vs H18) of the DEG of dre04260 cardiac muscle contraction (A-C) and dre04142 lysosome (D- F) KEGG pathways. (TIFF 9695 kb)

Additional file 6: Figure S2. Differentially expressed genes involved in the cardiac muscle contraction pathway. Color scale indicates the log transformed fold change (H18/CON) of differentially expressed genes. Significantly downregulated genes are shown in green. Significantly upregulated genes are shown in red. KEGG Pathway: dre04260. (TIFF 57 kb)

Additional file 7: Figure S3. Differentially expressed genes involved in the calcium signaling pathway. Color scale indicates the log transformed fold change (H18/CON) of differentially expressed genes. Significantly downregulated genes are shown in green. Significantly upregulated genes are shown in red. KEGG Pathway: dre04020. (TIFF 73 kb)

Additional file 8: Figure S4. Differentially expressed genes involved in the lysosome pathway. Color scale indicates the log transformed fold change (H18/CON) of differentially expressed genes. Significantly downregulated genes are shown in green. Significantly upregulated genes are shown in red. KEGG Pathway: dre04142. (TIFF 115 kb)

Additional file 9: Figure S5. Differentially expressed genes involved in the pyruvate metabolism pathway. Color scale indicates the log transformed fold change (H18/CON) of differentially expressed genes. Significantly downregulated genes are shown in green. Significantly upregulated genes are shown in red. KEGG Pathway: dre00620. (TIFF 80 kb)

Additional file 10: Figure S6. Differentially expressed genes involved in the oxidative phosphorylation pathway. Color scale indicates the log transformed fold change (H18/CON) of differentially expressed genes. Significantly downregulated genes are shown in green. Significantly upregulated genes are shown in red. KEGG Pathway: dre00190. (TIFF 222 kb)

Additional file 11: Figure S7. Differentially expressed genes involved in citrate cycle (TCA cycle) pathway. Color scale indicates the log transformed fold change (H18/CON) of differentially expressed genes. Significantly downregulated genes are shown in green. Significantly upregulated genes are shown in red. KEGG Pathway: dre00020. (TIFF 64 kb)

Additional file 12: Figure S8. Differentially expressed genes involved in the 2-oxocarboxylic acid metabolism pathway. Color scale indicates the log transformed fold change (H18/CON) of differentially expressed genes. Significantly downregulated genes are shown in green. Significantly upregulated genes are shown in red. KEGG Pathway: dre01210. (TIFF 103 kb)

Additional file 13: Figure S9. Differentially expressed genes involved in the glycolysis/gluconeogenesis pathway. Color scale indicates the log transformed fold change (H18/CON) of differentially expressed genes. Significantly downregulated genes are shown in green. Significantly upregulated genes are shown in red. KEGG Pathway: dre00010. (TIFF 69 kb)

Additional file 14: Figure S10. Differentially expressed genes involved in the carbon metabolism pathway. Color scale indicates the log transformed fold change (H18/CON) of differentially expressed genes. Significantly downregulated genes are shown in green. Significantly upregulated genes are shown in red. KEGG Pathway: dre01200. (TIFF 113 kb)

Additional file 15: Figure S11. Differentially expressed genes involved in the MAPK signaling pathway. Color scale indicates the log transformed fold change (H18/CON) of differentially expressed genes. Significantly downregulated genes are shown in green. Significantly upregulated genes are shown in red. Kegg Pathway: dre04010. (TIFF 105 kb)

Additional file 16: Figure S12. MAPK signaling KEGG pathway gene expression. Fold change bar plots (A, C, E) and gene expression dot plots (B, D, F) of the DEG of the dre04010 MAPK signaling pathway in the CON vs H9 (A, B), CON vs H18 (C, D) and H9 vs H18 (E, F). (TIFF 24685 kb)

Additional file 17: Figure S13. GO enrichment analysis of differentially expressed genes (DEG). Related to Fig. 3. Distribution of significantly perturbed GO terms in the three pair-wise comparisons (A). Significantly downregulated GO terms are shown in green. Significantly upregulated GO terms are shown in red. Cutoff for significance was q < 0.1 according to default values and parameters of the R package “gage” v2.22.0. Venn diagram showing overlap of perturbed GO terms between CON, H9, and H18 sample comparisons (B). Fold change (H18/CON) of the DEG of the GO:00422692 muscle cell differentiation (C), GO:0016052 carbohydrate catabolic process (D), and GO:005764 lysosome (E) GO terms. (TIFF 10813 kb)

Additional file 18: Table S4. Significantly perturbed GO terms. Terms are listed according to the composite log scaled expression of all genes assigned a given GO term. Significantly downregulated GO terms are shown in green. Significantly upregulated GO terms are shown in red. Cutoff for significance was q < 0.1 according to default values and parameters of the R package “gage” v2.22.0 (XLSX 77 kb)

Additional file 19: Table S5. PANTHER enrichment test analysis of profile “A: Progressive Downregulation” (clusters 1/3/7). Cutoff for significance was *P* < 0.05. (XLSX 10 kb)

Additional file 20: Table S6. PANTHER enrichment test analysis of profile “B: Persistent Downregulation” (clusters 4/10/11). Cutoff for significance was *P* < 0.05. (XLSX 11 kb)

Additional file 21: Table S7. PANTHER enrichment test analysis of profile “C: Persistent Upregulation” (clusters 2/5/13/14). Cutoff for significance was *P* < 0.05. (XLSX 14 kb)

Additional file 22: Table S8. PANTHER enrichment test analysis of profile “D: Delayed Downregulation” (clusters 6/17). Cutoff for significance was *P* < 0.05. (XLSX 10 kb)

Additional file 23: Table S9. PANTHER enrichment test analysis of profile “F: Transient Upregulation” (cluster 9). Cutoff for significance was *P* < 0.05. (XLSX 11 kb)

Additional file 24: Table S10. PANTHER enrichment test analysis of profile “G: Late Upregulation” (cluster 12). Cutoff for significance was *P* < 0.05. (XLSX 10 kb)

Additional file 25: Figure S15. Morpholino knockdown of gene expression. Related to Fig. 5. Craniectomy was performed to visualize EGFP-labeled muscle at 8 dpi. Brain was removed to allow visualization of the skull base (*) Microinjected MOs are detected throughout the entire regenerating muscle, including the distal ends (white arrow). Most MOs targeting specific mRNA decreased muscle regeneration at 8 dpi. Control MO (shown again for comparative purposes), *ezh2* and *suz12a* MO injected fish are shown, Fig. 5 shows the *fn1a* MO. (TIFF 23944 kb)

Additional file 26: Figure S14. Enlarged heatmap. Related to Fig. 1. Gene symbols are shown (left) and color scale (right) indicates gene expression (FPKM). DEG are ordered according to numeric clusters from Fig. 4, represented as a color bar on the left side (color code is shown in Fig. 4 over each cluster plot), and listed in Additional file 3: Table S2. (TIFF 11237 kb)

Additional file 27: Figure S16. Cell cycle inhibition and EOM regeneration. (A) Bortezomid is a proteasome inhibitor that blocks cell cycle [66, 67]. Fish were treated with 5 μM bortozemid by 2000X dilution of a 10 mM DMSO stock in fish water, same DMSO concentration was used in control group. Cell proliferation at 24 hpi was analyzed by intraperitoneal EdU injection as described before. (B) 5-fluorouracil (5-Flu) is a pyrimidine analog that blocks cell cycle through irreversible inhibition of thymidylate synthase [68]. Fish were injected with 10 mM 5-Flu diluted in PBS, PBS injections were used as control. Cell proliferation at 24 hpi was analyzed by intraperitoneal EdU injection as described. Both treatments (bortezomib, A, or 5-Flu, B) effectively blocked cell cycle progression (no EdU staining) in the regenerating muscle. Note that the mesenchymal transition did not progress and the injured muscle retained its typical sarcomere architecture, as evidenced by DIC microscopy. Pictures are representative examples of 5 fish per group. (TIFF 19117 kb)

Additional file 28: Figure S17. Diagram of zebrafish EOMs. Sketch of a zebrafish head coronal section depicting the extraocular muscles visualized by the craniectomy technique (A). The dashed box in A approximately shows the picture used for regeneration assessment. Diagram of a regeneration assessment picture showing injured and uninjured muscles (B). Formula used to calculate the relative growth of the injured muscle (C). (TIFF 10948 kb)

## Abbreviations

Dpi: days post injury
EdU: 5-ethynyl-2′-deoxyuridine
EOM: Extraocular muscle
Hpi: Hours post injury
LR: Lateral rectus
MO: Morpholino oligonucleotide
