# Supplementary figures and images for: Temporally distinct transcriptional regulation of myocyte dedifferentiation and Myofiber growth during muscle regeneration
Source: BMC Genomics. 2017 Nov 9;18:854. doi: 10.1186/s12864-017-4236-y (PMC5680785; doi:10.1186/s12864-017-4236-y)

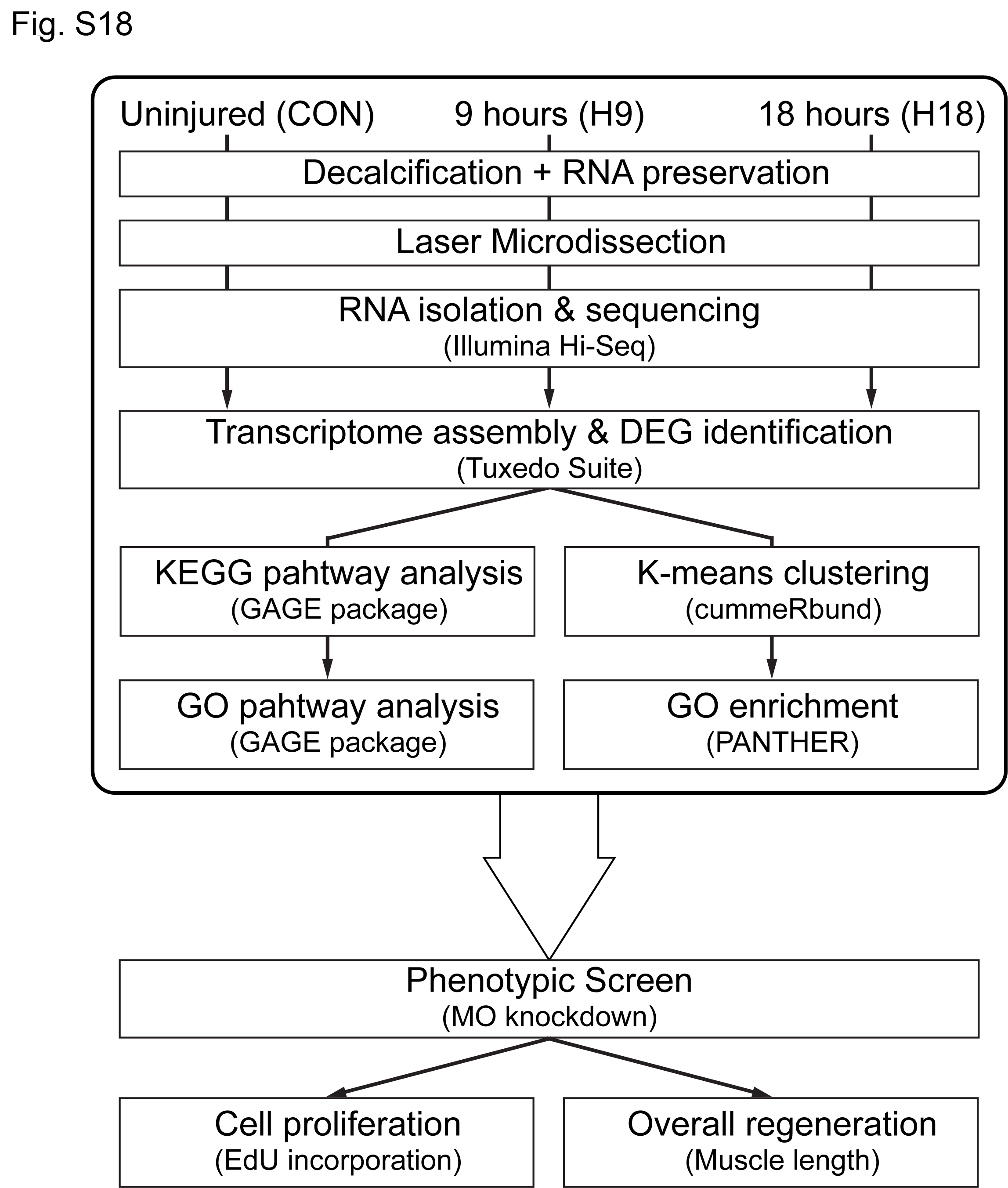

Supplement: Supplementary file 1 — Diagram of the strategy of the present study. RNA was isolated and sequenced from laser microdissected adult zebrafish LR muscle tissue at multiple time points post injury. Transcriptome assembly and DEG identification was performed using the Tuxedo Suite. DEG functional classification was concurrently performed via separate strategies using either gene set enrichment analysis (left: KEGG pathway, GO term analysis [GAGE]) or expression profile-based clustering (right: K-means clustering [cummeRbund] and GO enrichment [Panther]). MO knockdown-based phenotypic screens included both cell proliferation and overall regeneration assays as readouts of DEG functional significance. (TIFF 18214 kb) [file 12864_2017_4236_MOESM1_ESM.tif]

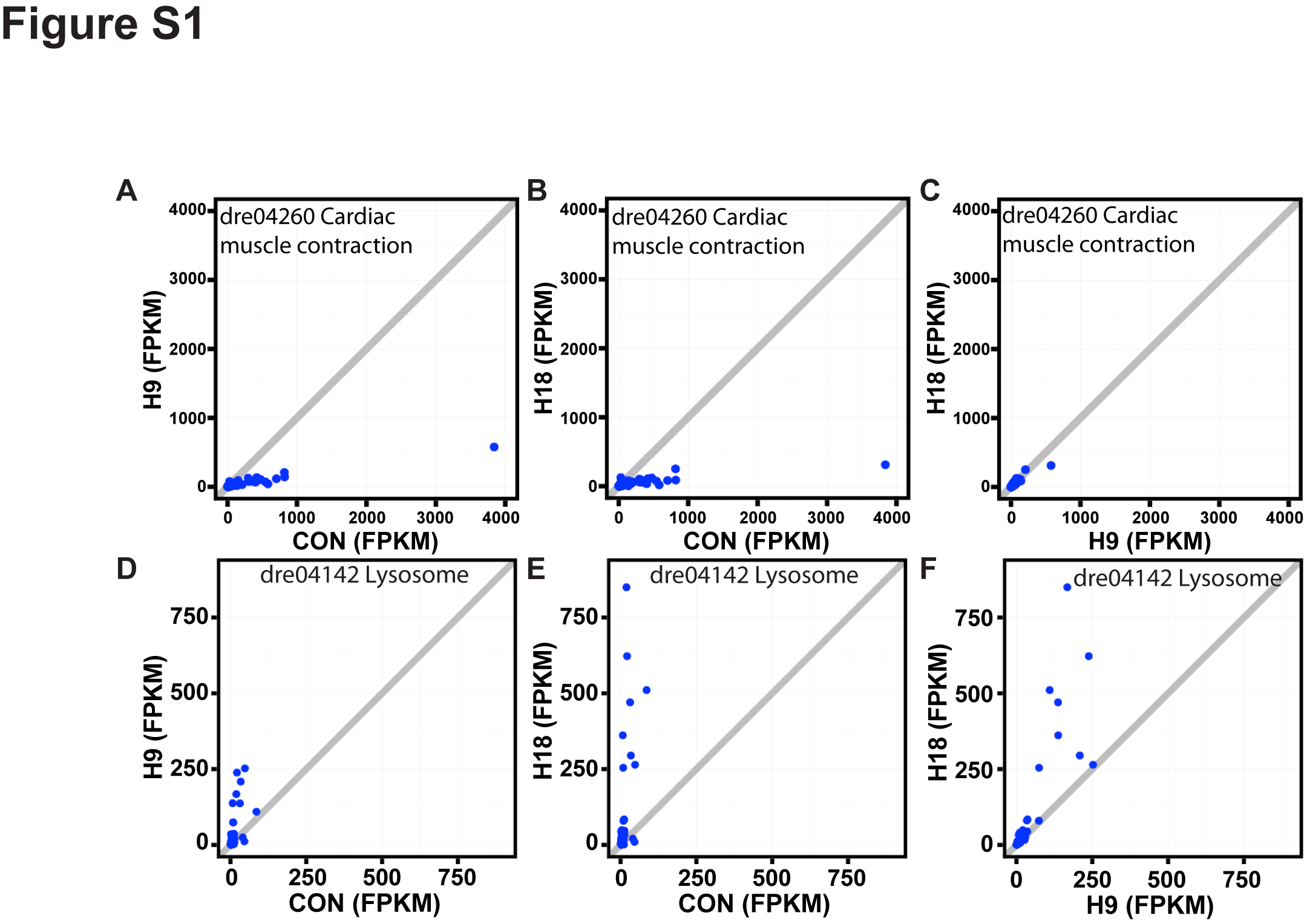

Supplement: Supplementary file 5 — Cardiac muscle contraction and lysosome KEGG pathway gene expression. Related to Fig. 2. Dot plot of gene expression (FPKM, CON vs H9, CON vs 18, H9 vs H18) of the DEG of dre04260 cardiac muscle contraction (A-C) and dre04142 lysosome (D- F) KEGG pathways. (TIFF 9695 kb) [file 12864_2017_4236_MOESM5_ESM.tif]

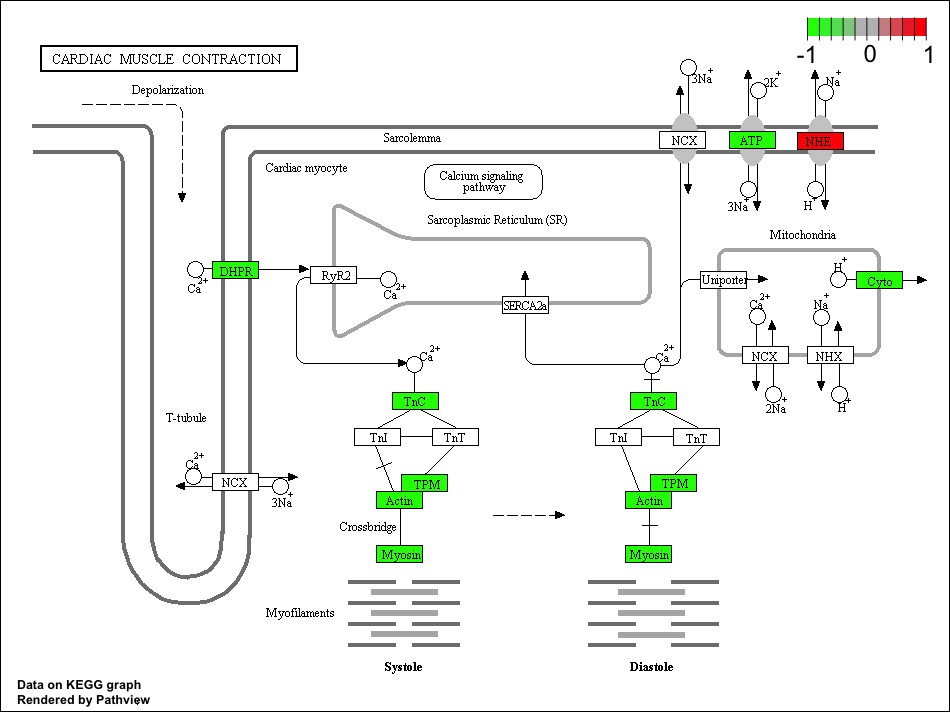

Supplement: Supplementary file 6 — Differentially expressed genes involved in the cardiac muscle contraction pathway. Color scale indicates the log transformed fold change (H18/CON) of differentially expressed genes. Significantly downregulated genes are shown in green. Significantly upregulated genes are shown in red. KEGG Pathway: dre04260. (TIFF 57 kb) [file 12864_2017_4236_MOESM6_ESM.tif]

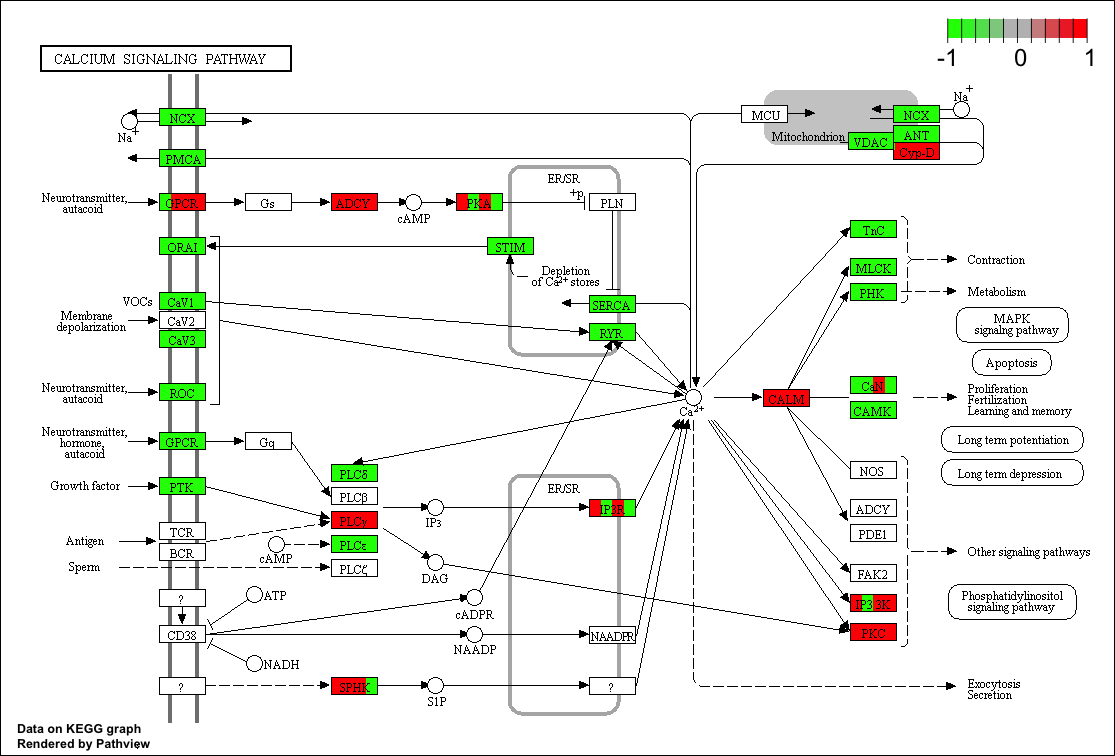

Supplement: Supplementary file 7 — Differentially expressed genes involved in the calcium signaling pathway. Color scale indicates the log transformed fold change (H18/CON) of differentially expressed genes. Significantly downregulated genes are shown in green. Significantly upregulated genes are shown in red. KEGG Pathway: dre04020. (TIFF 73 kb) [file 12864_2017_4236_MOESM7_ESM.tif]

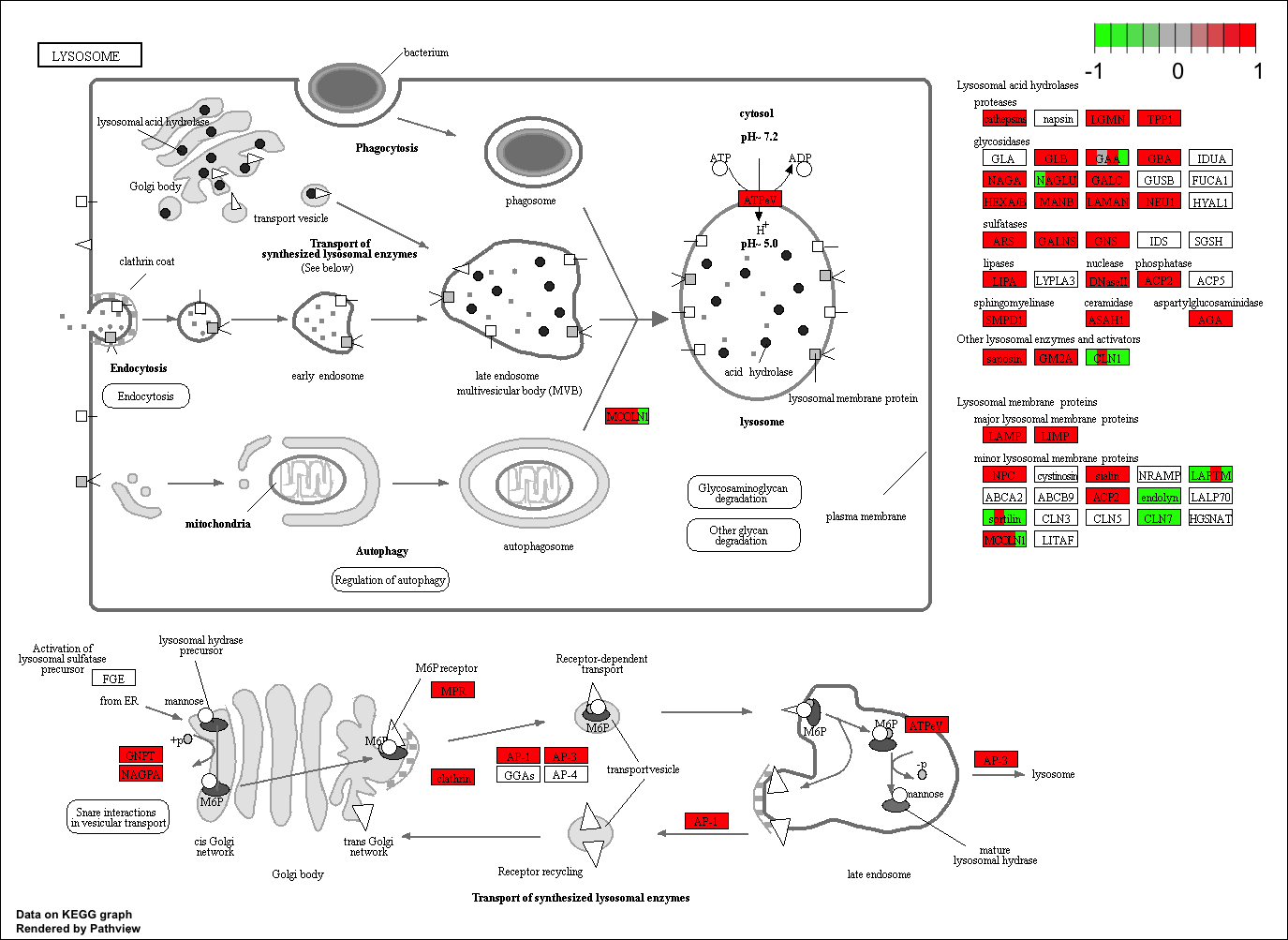

Supplement: Supplementary file 8 — Differentially expressed genes involved in the lysosome pathway. Color scale indicates the log transformed fold change (H18/CON) of differentially expressed genes. Significantly downregulated genes are shown in green. Significantly upregulated genes are shown in red. KEGG Pathway: dre04142. (TIFF 115 kb) [file 12864_2017_4236_MOESM8_ESM.tif]

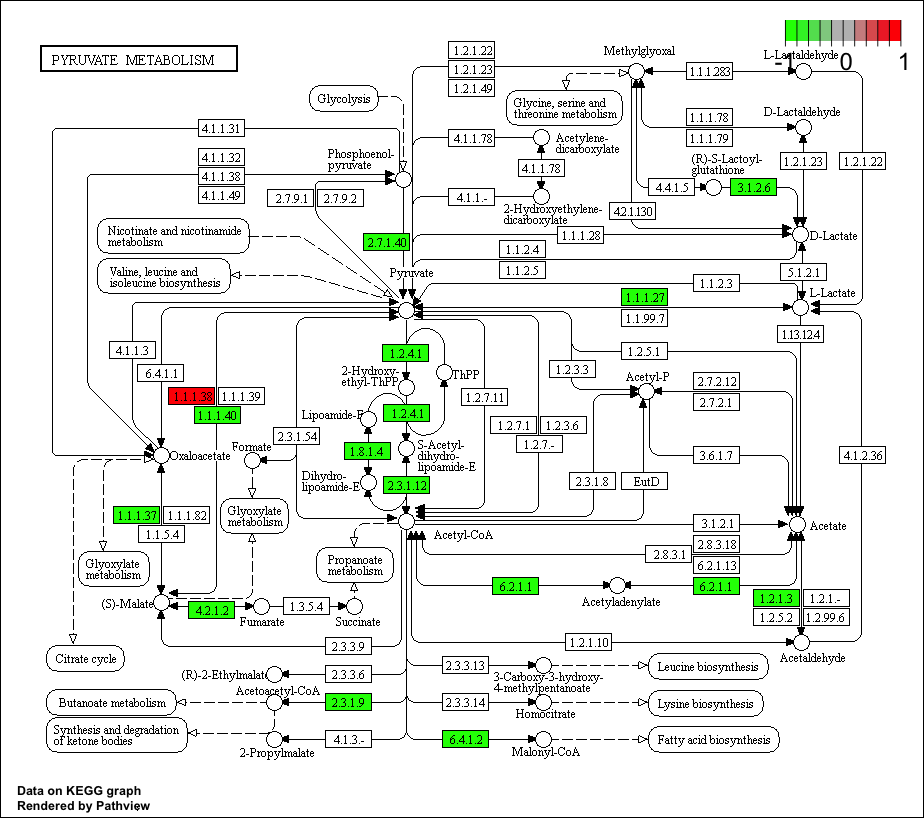

Supplement: Supplementary file 9 — Differentially expressed genes involved in the pyruvate metabolism pathway. Color scale indicates the log transformed fold change (H18/CON) of differentially expressed genes. Significantly downregulated genes are shown in green. Significantly upregulated genes are shown in red. KEGG Pathway: dre00620. (TIFF 80 kb) [file 12864_2017_4236_MOESM9_ESM.tif]

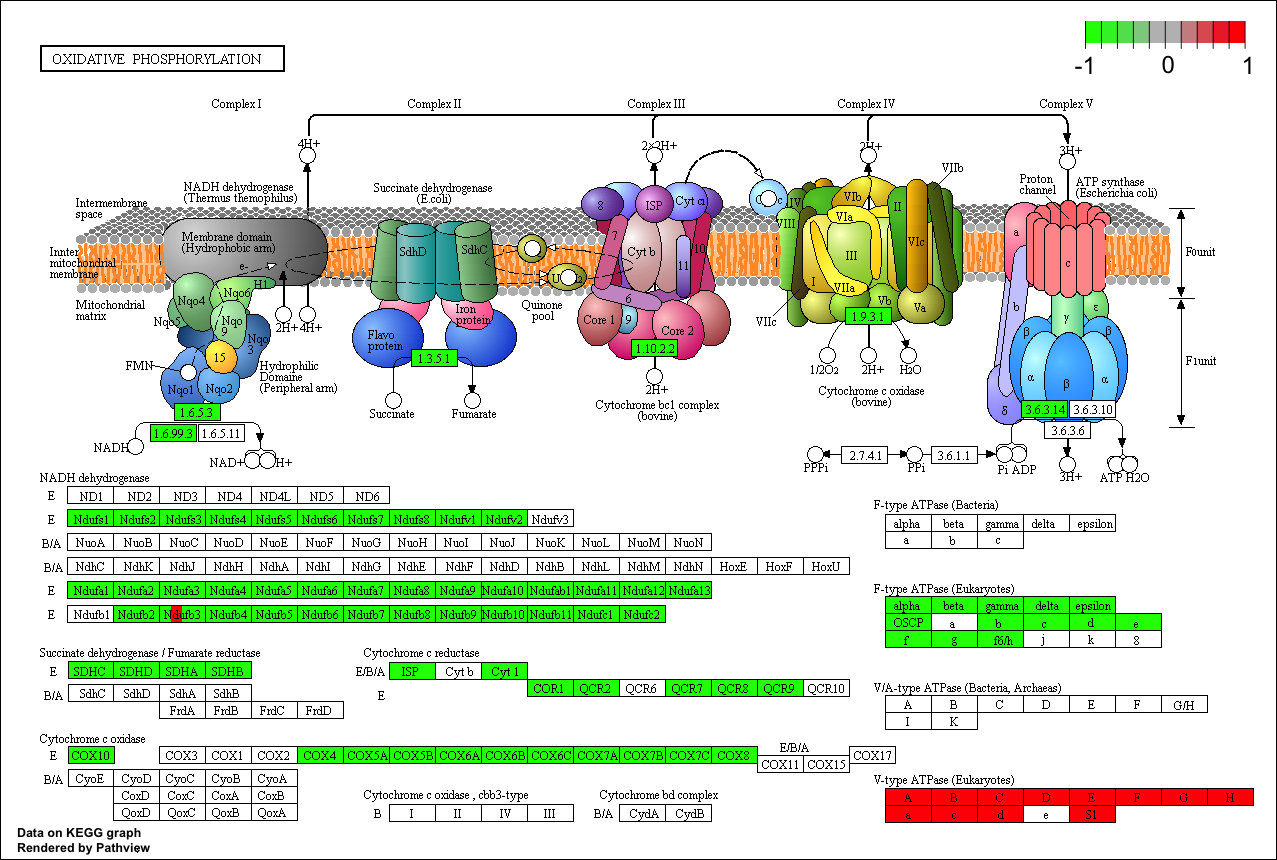

Supplement: Supplementary file 10 — Differentially expressed genes involved in the oxidative phosphorylation pathway. Color scale indicates the log transformed fold change (H18/CON) of differentially expressed genes. Significantly downregulated genes are shown in green. Significantly upregulated genes are shown in red. KEGG Pathway: dre00190. (TIFF 222 kb) [file 12864_2017_4236_MOESM10_ESM.tif]

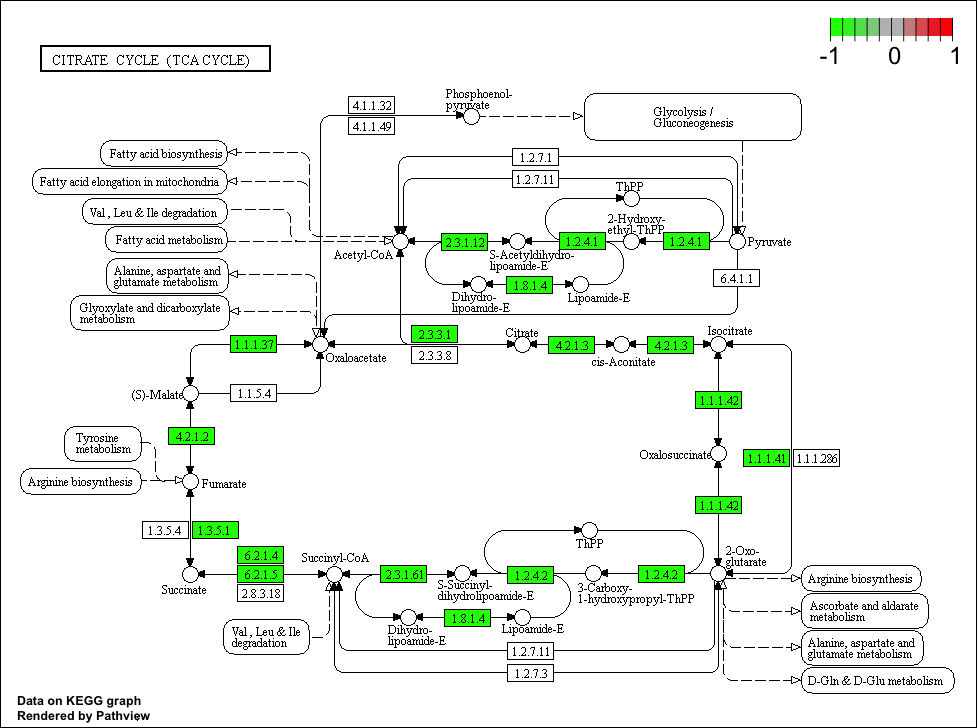

Supplement: Supplementary file 11 — Differentially expressed genes involved in citrate cycle (TCA cycle) pathway. Color scale indicates the log transformed fold change (H18/CON) of differentially expressed genes. Significantly downregulated genes are shown in green. Significantly upregulated genes are shown in red. KEGG Pathway: dre00020. (TIFF 64 kb) [file 12864_2017_4236_MOESM11_ESM.tif]

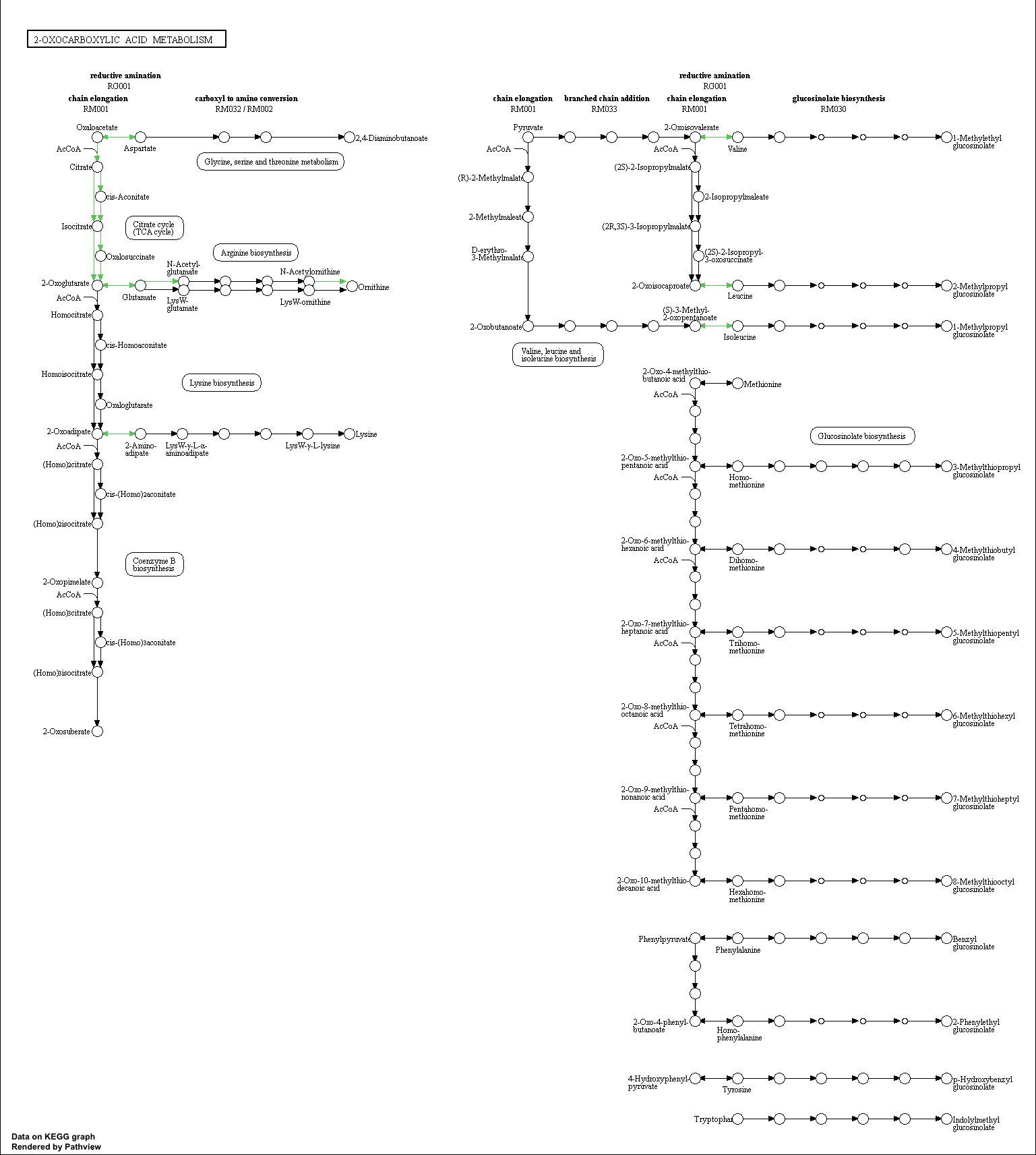

Supplement: Supplementary file 12 — Differentially expressed genes involved in the 2-oxocarboxylic acid metabolism pathway. Color scale indicates the log transformed fold change (H18/CON) of differentially expressed genes. Significantly downregulated genes are shown in green. Significantly upregulated genes are shown in red. KEGG Pathway: dre01210. (TIFF 103 kb) [file 12864_2017_4236_MOESM12_ESM.tif]

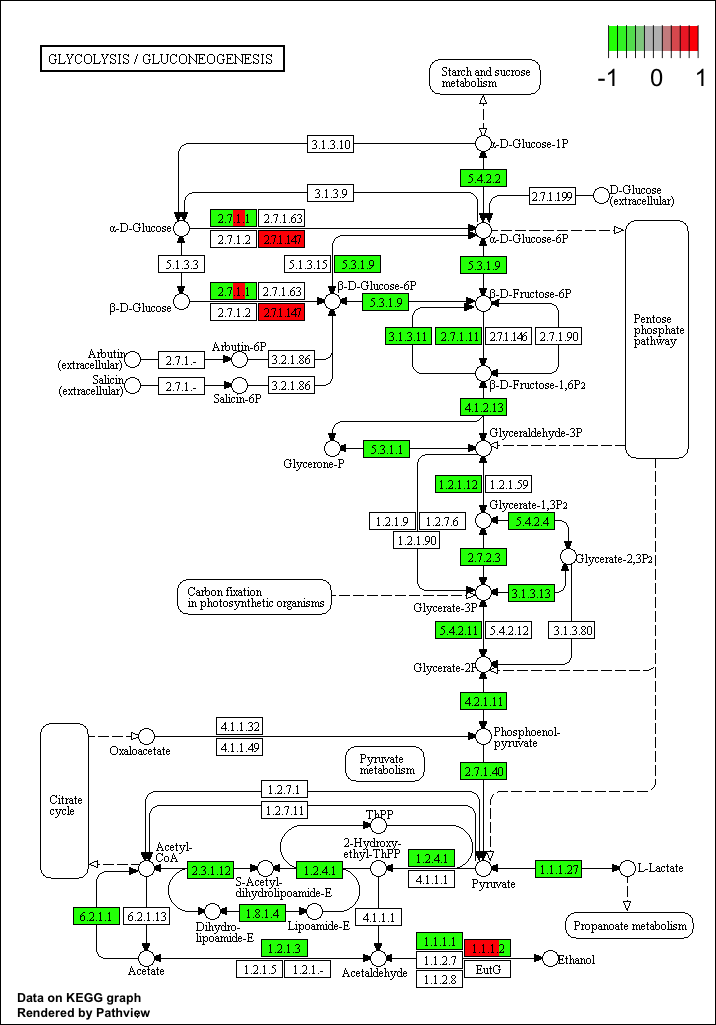

Supplement: Supplementary file 13 — Differentially expressed genes involved in the glycolysis/gluconeogenesis pathway. Color scale indicates the log transformed fold change (H18/CON) of differentially expressed genes. Significantly downregulated genes are shown in green. Significantly upregulated genes are shown in red. KEGG Pathway: dre00010. (TIFF 69 kb) [file 12864_2017_4236_MOESM13_ESM.tif]

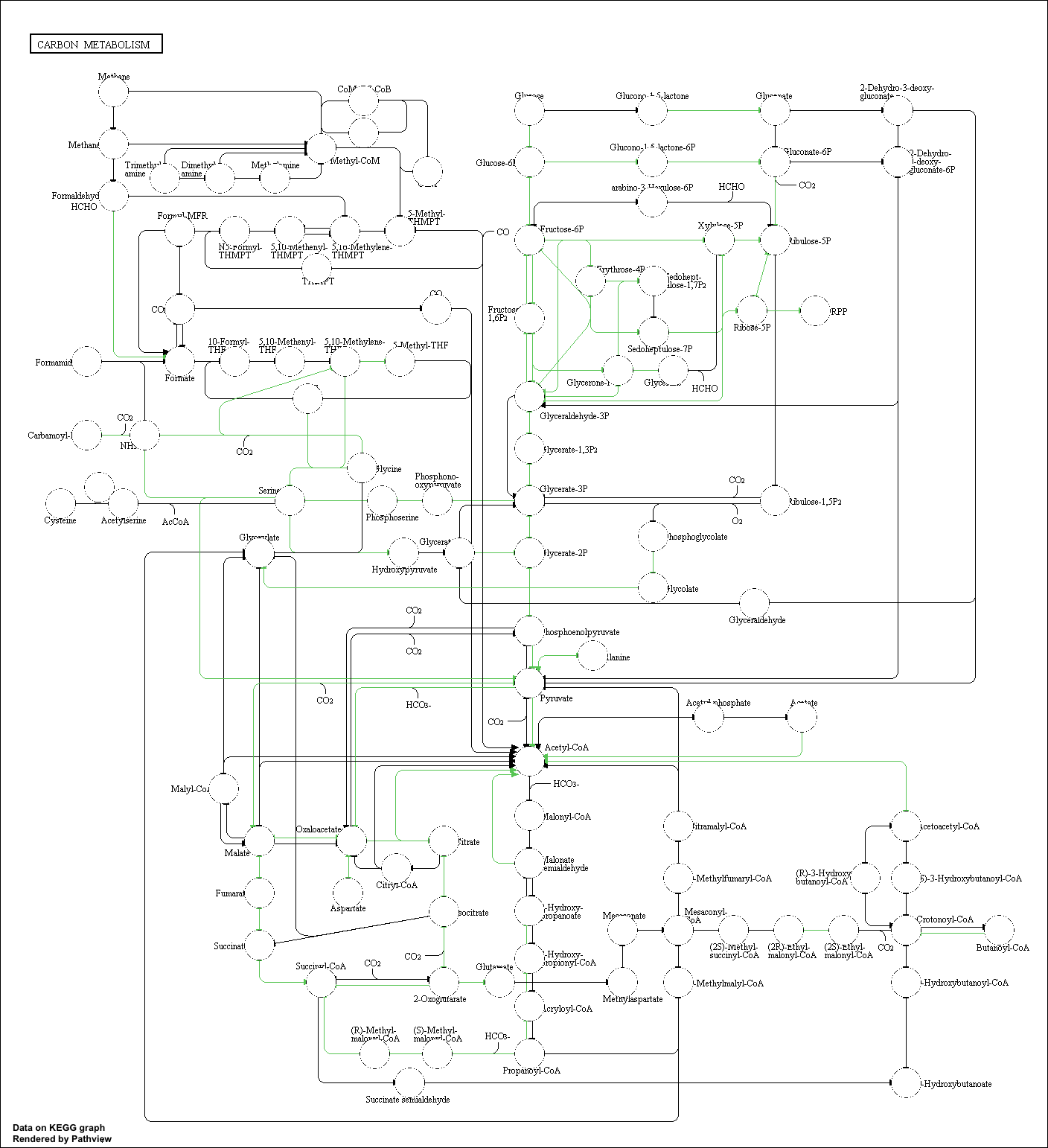

Supplement: Supplementary file 14 — Differentially expressed genes involved in the carbon metabolism pathway. Color scale indicates the log transformed fold change (H18/CON) of differentially expressed genes. Significantly downregulated genes are shown in green. Significantly upregulated genes are shown in red. KEGG Pathway: dre01200. (TIFF 113 kb) [file 12864_2017_4236_MOESM14_ESM.tif]

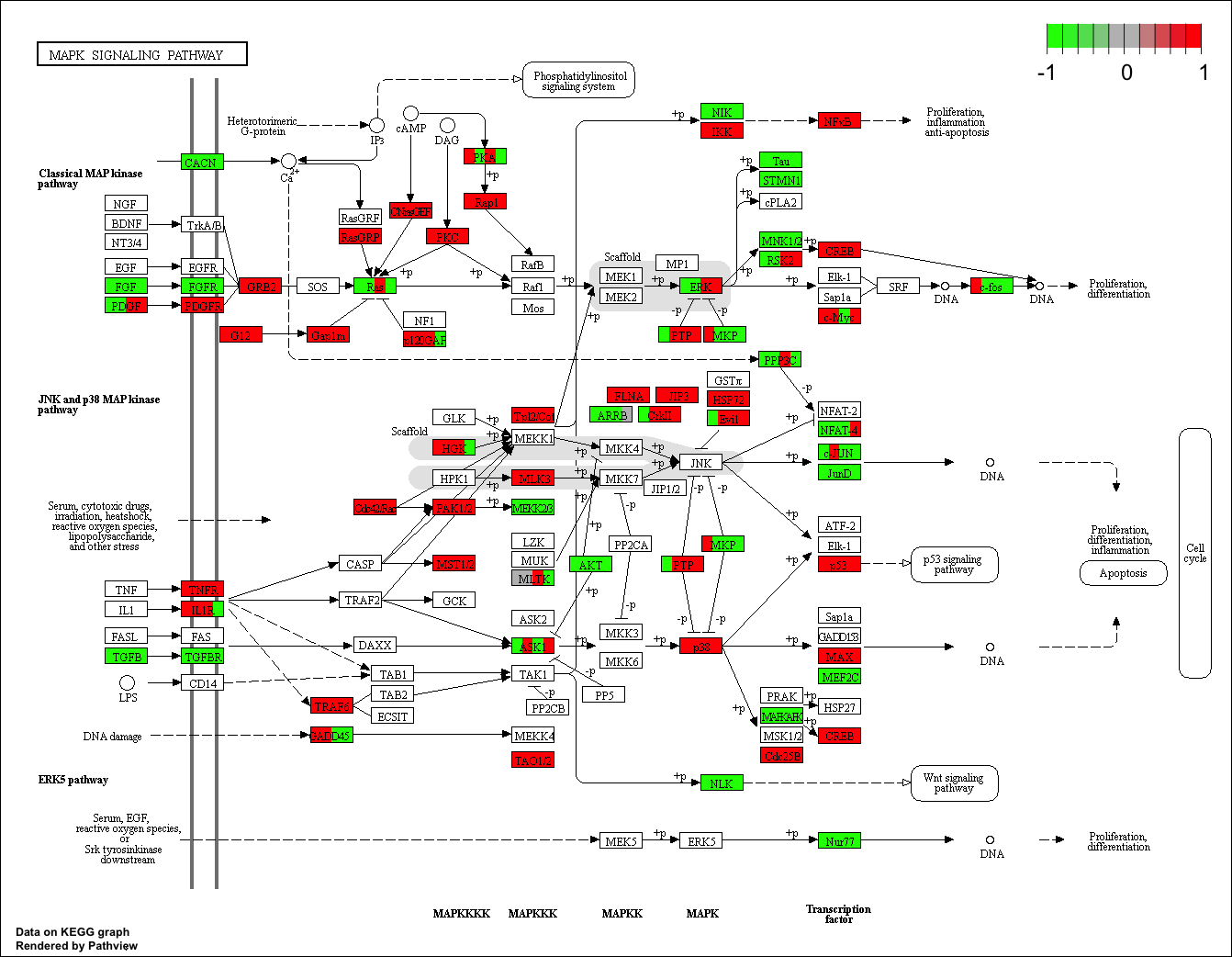

Supplement: Supplementary file 15 — Differentially expressed genes involved in the MAPK signaling pathway. Color scale indicates the log transformed fold change (H18/CON) of differentially expressed genes. Significantly downregulated genes are shown in green. Significantly upregulated genes are shown in red. Kegg Pathway: dre04010. (TIFF 105 kb) [file 12864_2017_4236_MOESM15_ESM.tif]

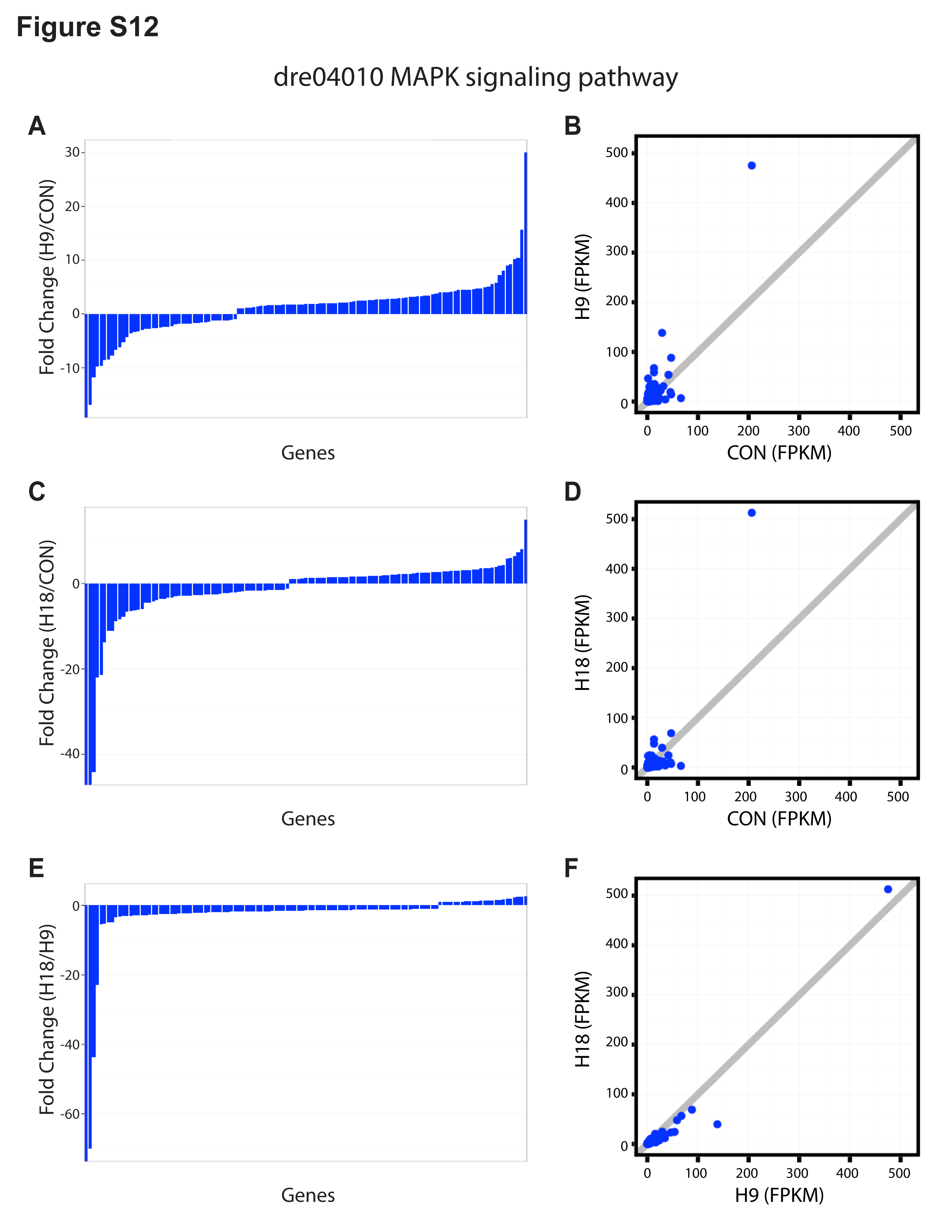

Supplement: Supplementary file 16 — MAPK signaling KEGG pathway gene expression. Fold change bar plots (A, C, E) and gene expression dot plots (B, D, F) of the DEG of the dre04010 MAPK signaling pathway in the CON vs H9 (A, B), CON vs H18 (C, D) and H9 vs H18 (E, F). (TIFF 24685 kb) [file 12864_2017_4236_MOESM16_ESM.tif]

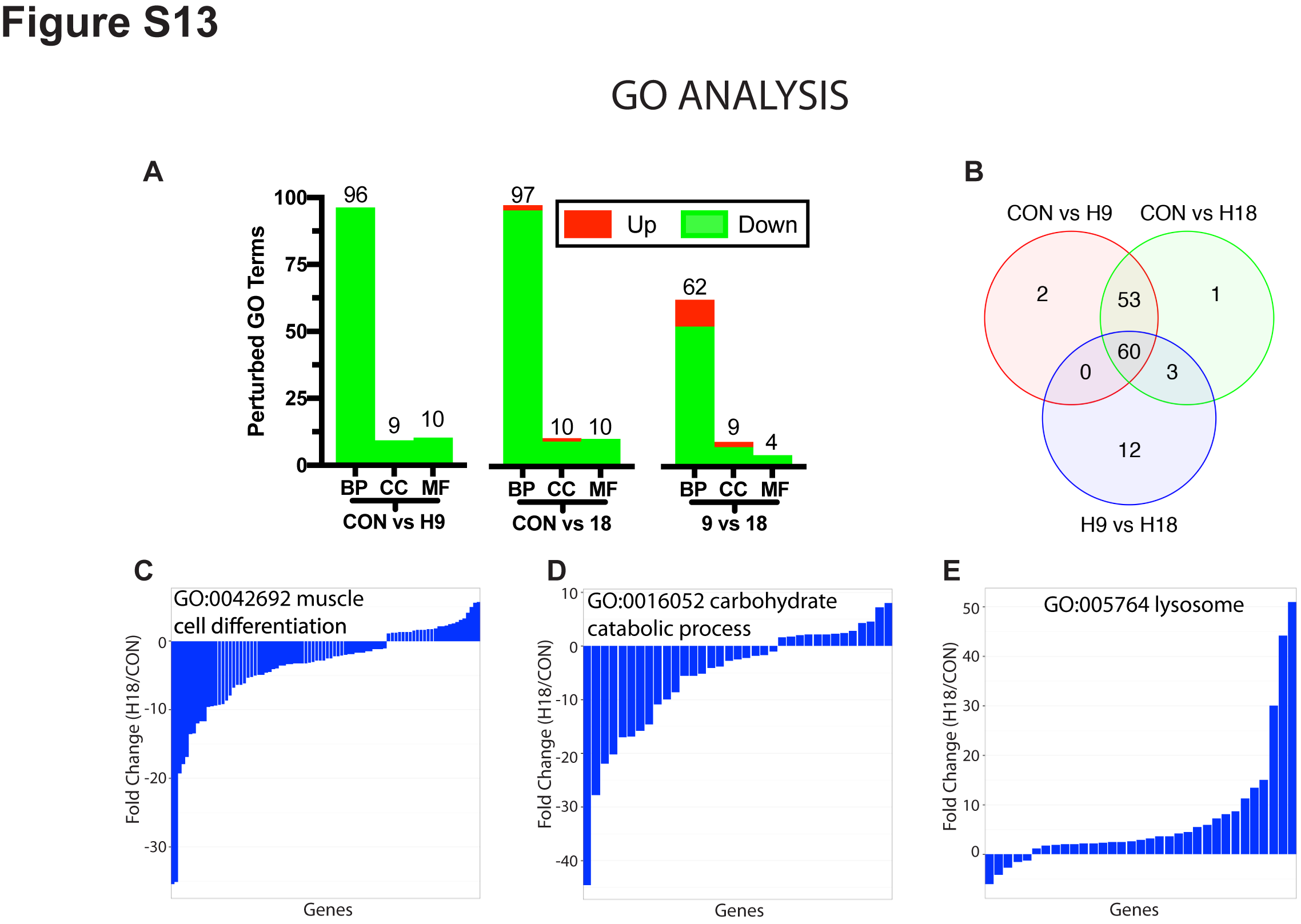

Supplement: Supplementary file 17 — GO enrichment analysis of differentially expressed genes (DEG). Related to Fig. 3. Distribution of significantly perturbed GO terms in the three pair-wise comparisons (A). Significantly downregulated GO terms are shown in green. Significantly upregulated GO terms are shown in red. Cutoff for significance was q < 0.1 according to default values and parameters of the R package “gage” v2.22.0. Venn diagram showing overlap of perturbed GO terms between CON, H9, and H18 sample comparisons (B). Fold change (H18/CON) of the DEG of the GO:00422692 muscle cell differentiation (C), GO:0016052 carbohydrate catabolic process (D), and GO:005764 lysosome (E) GO terms. (TIFF 10813 kb) [file 12864_2017_4236_MOESM17_ESM.tif]

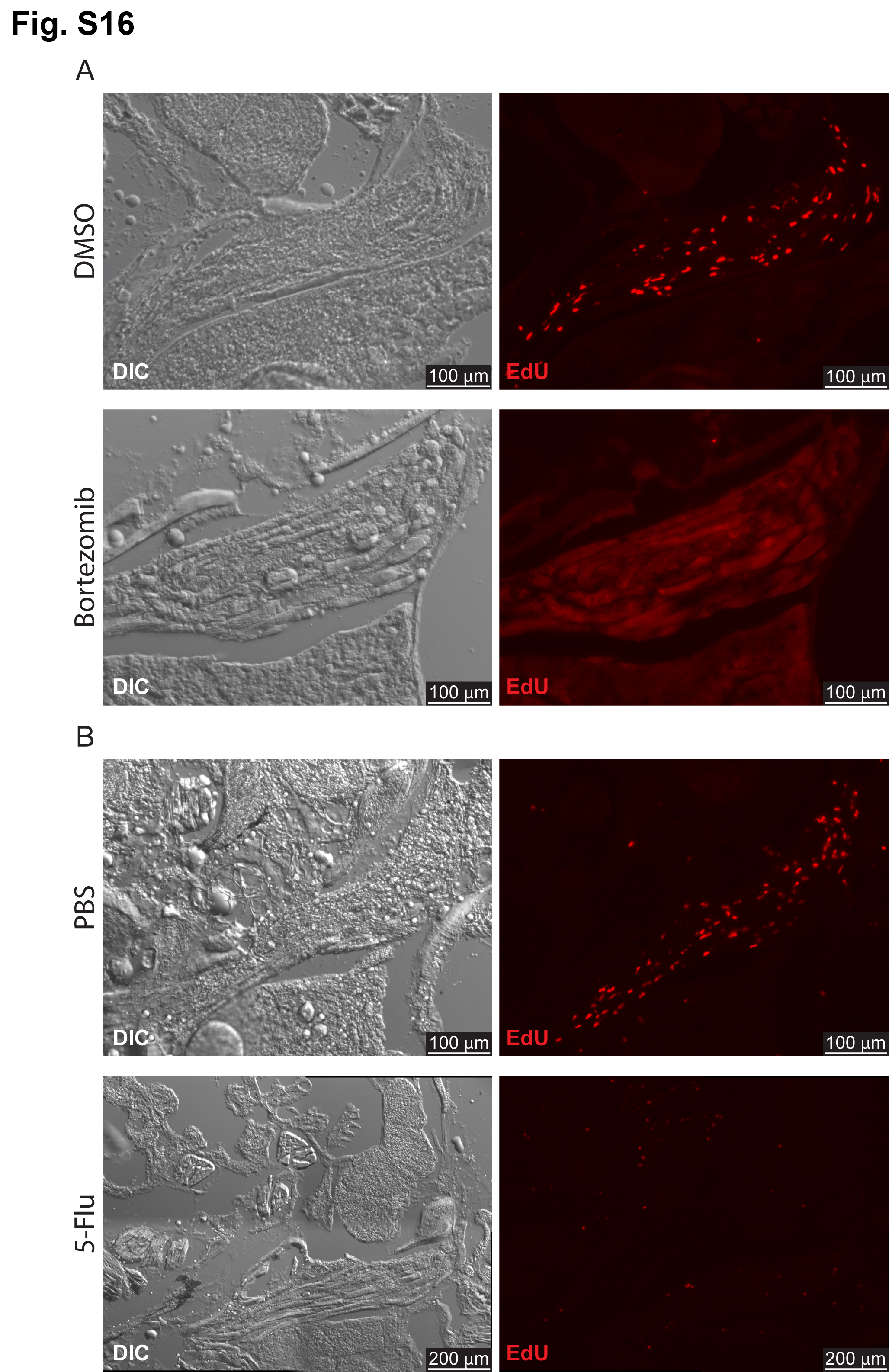

Supplement: Supplementary file 27 — Cell cycle inhibition and EOM regeneration. (A) Bortezomid is a proteasome inhibitor that blocks cell cycle [66, 67]. Fish were treated with 5 μM bortozemid by 2000X dilution of a 10 mM DMSO stock in fish water, same DMSO concentration was used in control group. Cell proliferation at 24 hpi was analyzed by intraperitoneal EdU injection as described before. (B) 5-fluorouracil (5-Flu) is a pyrimidine analog that blocks cell cycle through irreversible inhibition of thymidylate synthase [68]. Fish were injected with 10 mM 5-Flu diluted in PBS, PBS injections were used as control. Cell proliferation at 24 hpi was analyzed by intraperitoneal EdU injection as described. Both treatments (bortezomib, A, or 5-Flu, B) effectively blocked cell cycle progression (no EdU staining) in the regenerating muscle. Note that the mesenchymal transition did not progress and the injured muscle retained its typical sarcomere architecture, as evidenced by DIC microscopy. Pictures are representative examples of 5 fish per group. (TIFF 19117 kb) [file 12864_2017_4236_MOESM27_ESM.tif]

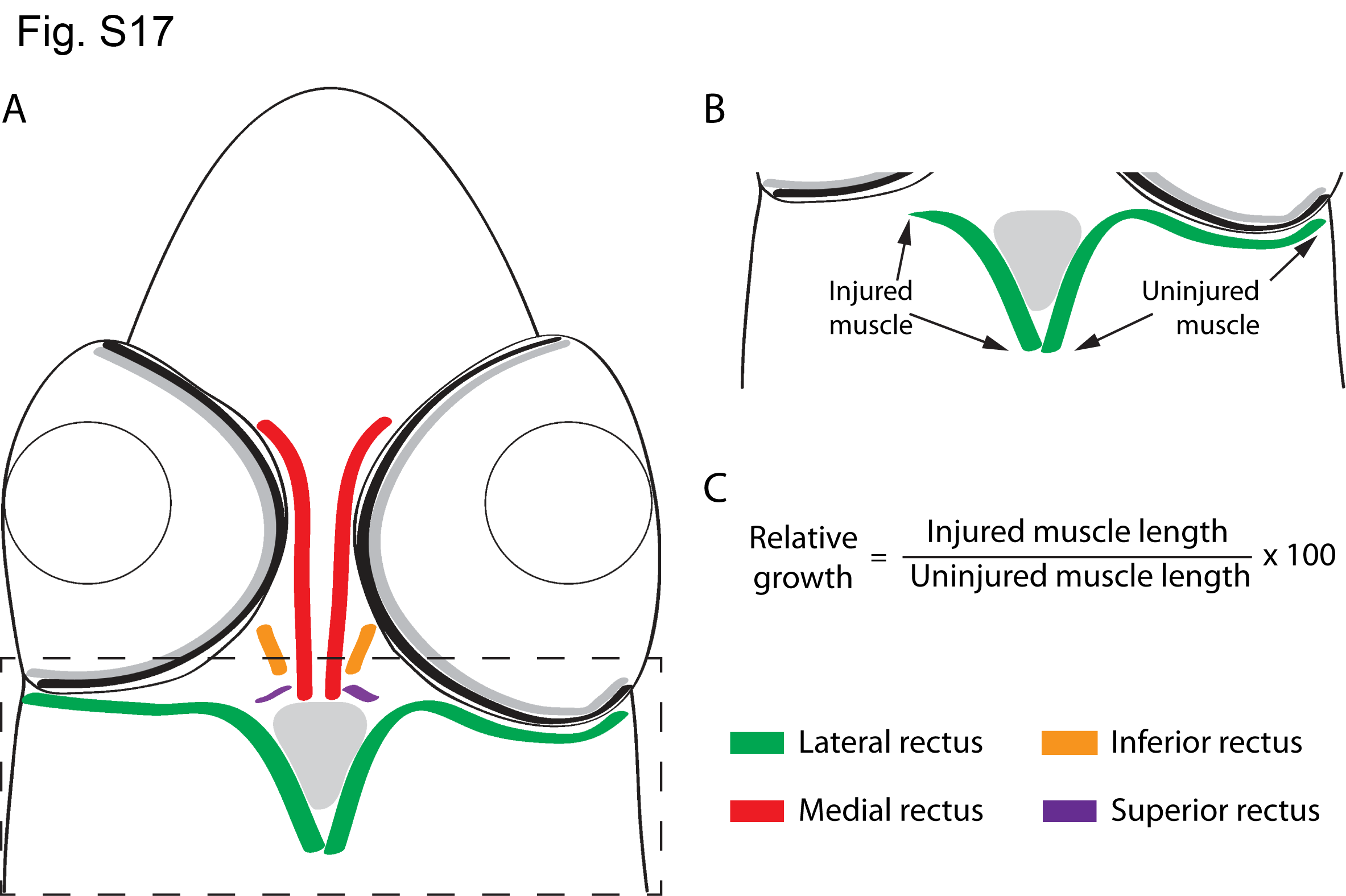

Supplement: Supplementary file 28 — Diagram of zebrafish EOMs. Sketch of a zebrafish head coronal section depicting the extraocular muscles visualized by the craniectomy technique (A). The dashed box in A approximately shows the picture used for regeneration assessment. Diagram of a regeneration assessment picture showing injured and uninjured muscles (B). Formula used to calculate the relative growth of the injured muscle (C). (TIFF 10948 kb) [file 12864_2017_4236_MOESM28_ESM.tif]
