# Supplementary material for: Temporally distinct transcriptional regulation of myocyte dedifferentiation and Myofiber growth during muscle regeneration
Source: BMC Genomics. 2017 Nov 9;18:854. doi: 10.1186/s12864-017-4236-y (PMC5680785; doi:10.1186/s12864-017-4236-y)
Supplement: Supplementary file 2 — Read counts and alignment rates. (DOCX 17 kb) [file 12864_2017_4236_MOESM2_ESM.docx]

**Table 1.** GO term annotations for gene expression profiles.

| Profile (cluster) | Gene number | Annotated genes | % |
| --- | --- | --- | --- |
|  |  |  |  |
|  |  |  |  |
| A (1/3/7) | 994 | 671 | 67.51 |
| B (4/10/11) | 1723 | 1296 | 75.22 |
| C (2/5/13/14) | 2172 | 1682 | 77.44 |
| D (6/17) | 477 | 309 | 64.78 |
| E (8) | 129 | 23 | 17.83 |
| F (9) | 648 | 476 | 73.46 |
| G (12) | 259 | 32 | 12.36 |
| H (15) | 42 | 14 | 33.33 |
| I (16) | 85 | 24 | 28.24 |
| J (18) | 67 | 11 | 16.42 |
